# Glucocorticoid-induced cell-derived matrix modulates transforming growth factor β2 signaling in human trabecular meshwork cells

**DOI:** 10.1038/s41598-020-72779-w

**Published:** 2020-09-24

**Authors:** Felix Yemanyi, Janice Vranka, Vijay Krishna Raghunathan

**Affiliations:** 1 Department of Basic Sciences, College of Optometry, University of Houston, Houston, TX USA; 2 Casey Eye Institute, Oregon Health and Science University, Portland, OR USA; 3 Department of Biomedical Engineering, Cullen College of Engineering, University of Houston, Houston, TX USA

**Keywords:** Cell biology, Cell signalling, Mechanisms of disease

## Abstract

Aberrant remodeling of trabecular meshwork (TM) extracellular matrix (ECM) may induce ocular hypertensive phenotypes in human TM (hTM) cells to cause ocular hypertension, via a yet unknown mechanism. Here, we show that, in the absence of exogenous transforming growth factor-beta2 (TGFβ2), compared with control matrices (VehMs), glucocorticoid-induced cell-derived matrices (GIMs) trigger non-Smad TGFβ2 signaling in hTM cells, correlated with overexpression/activity of structural ECM genes (fibronectin, collagen IV, collagen VI, myocilin), matricellular genes (connective tissue growth factor [CTGF], secreted protein, acidic and rich in cysteine), crosslinking genes/enzymes (lysyl oxidase, lysyl oxidase-like 2–4, tissue transglutaminase-2), and ECM turnover genes/enzymes (matrix metalloproteinases-MMP2,14 and their inhibitors-TIMP2). However, in the presence of exogenous TGFβ2, VehMs and GIMs activate Smad and non-Smad TGFβ2 signaling in hTM cells, associated with overexpression of α-smooth muscle actin (α-SMA), and differential upregulation of aforementioned ECM genes/proteins with new ones emerging (collagen-I, thrombospondin-I, plasminogen activator inhibitor, MMP1, 9, ADAMTS4, TIMP1); with GIM-TGFβ2-induced changes being mostly more pronounced. This suggests dual glaucomatous insults potentiate profibrotic signaling/phenotypes. Lastly, we demonstrate type I TGFβ receptor kinase inhibition abrogates VehM-/GIM- and/or TGFβ2-induced upregulation of α-SMA and CTGF. Collectively, pathological TM microenvironments are sufficient to elicit adverse cellular responses that may be ameliorated by targeting TGFβ2 pathway.

## Introduction

Ocular hypertension predominantly results from obstruction to aqueous humor outflow due to changes in the juxtacanalicular (JCT) portion of the trabecular meshwork (TM) and its interface with the inner wall endothelium of Schlemm’s canal (SC)^1–3^. Although multiple factors have been implicated in the changes at the JCT to hinder aqueous outflow^4–7^, aberrant extracellular matrix (ECM) remodeling culminating in altered stiffness has emerged as a significant risk factor^8–16^. In the TM, a pathologically remodeled ECM may likely have an effect through its interaction with resident cells and/or adjacent SC cells and contribute to disease progression^1,3,13^. However, the mechanistic basis for such a phenomenon is poorly understood. This is largely due to technical limitations in experimental animal models wherein dissecting out the interactions between multiple ECM components, either alone or in concert with exogenous stimuli to influence cellular fate is difficult. Thus, to study bi-directional interactions between cells and their ECM requires robust in vitro models. Cell-derived matrices^17,18^ thus present as indispensable tools to investigate the mechanistic basis of pathology, and more importantly, different matrices may be generated with various stimuli. For example, our group has documented that matrices derived after glucocorticoid treatment (GIMs) replicate many features of glaucomatous matrices and are potent enough to alter cell fate^11,13^. Also, compared with other in vitro hydrogel-based ECM-mimicking substrates^19–22^, GIMs better reflect the complexity of a diseased TM ECM^11,23^.

Transforming growth factor beta (TGFβ) signaling is well-implicated in glaucoma^24–26^ with its ligand, TGFβ2, elevated in the aqueous humor of primary open angle glaucoma patients^27,28^, and is overexpressed with dexamethasone/steroid induction^29,30^. TGFβ2 binds to its receptor complex (type I and type II) and subsequently activates the Smad- (Smad2 and Smad3)^31^ and non-Smad-dependent (extracellular signal regulated kinase [ERK], P38, c-Jun N-terminal kinases [JNK], RhoA, and Rho-associated protein kinase [ROCK])^32^ signaling pathways to induce ocular hypertension^24^, concurrent with pathological changes in the actin cytoskeleton (for example, stress fibers and crosslinked actin networks)^33^ and ECM proteins^16,25,34–37^. Since TGFβ2- and DEX-induced ocular hypertensive phenotypes are predicated on similar changes in actin cytoskeleton^33,38–40^, ECM^16,41–43^, and proteomic^44^ outcomes, there could be crosstalk between their respective signaling pathways. Indeed, Kasetti and colleagues^29^ recently showed that Smad-dependent TGFβ signaling pathways have crucial roles in DEX-induced ocular hypertension. However, in the absence of exogenous DEX, whether pathologic matrices such as GIMs can intrinsically modulate Smad- and non-Smad-dependent TGFβ signaling pathways in human TM (hTM) cells remains to be demonstrated. Previously, our group^11^ demonstrated that, GIMs harbor significantly increased levels of profibrotic molecules like TGFβ2, connective tissue growth factor (CTGF)^45^, thrombospondin 1 (TSP1)^46^, secreted protein acidic and rich in cysteine (SPARC)^35^, and antagonists of the Wnt pathway^47^; all of which can activate TGFβ signaling.

Therefore, in this study, we first determined the intrinsic ability of GIMs to modulate Smad and non-Smad TGFβ2 signaling pathways in hTM cells. Next, since ocular hypertensive phenotypes are typically multifactorial, we investigated the effect of the interaction between GIMs and exogenous TGFβ2 on this pathway. Lastly, we explored whether inhibition of type I TGFβ receptor kinase ameliorates the deleterious effects induced by GIMs. A portion of the findings in this study has been reported elsewhere (Yemanyi F, et al. *IOVS* 2019; 60: ARVO E-abstract 5146)^48^.

## Results

### Resident hTM cells were successfully removed from their deposited VehMs and GIMs

Vehicle control- (VehMs) and glucocorticoid-induced matrices (GIMs) were confirmed to be free of cytosolic and nuclear contamination by immunocytochemistry. Morphological or topographical differences in collagen IV observed between groups (VehM and GIM) (Supplementary Fig. S1A), while absence of labeling for F-actin and DAPI confirmed successful denudation of resident hTM cells. Supplementary Fig. S1B shows subsequent recellularization of VehMs and GIMs labeled for collagen IV and fibronectin, respectively.

### GIMs activated Smad TGFβ2 pathways only in the presence of exogenous TGFβ2 in hTM cells

**Figure 1:**
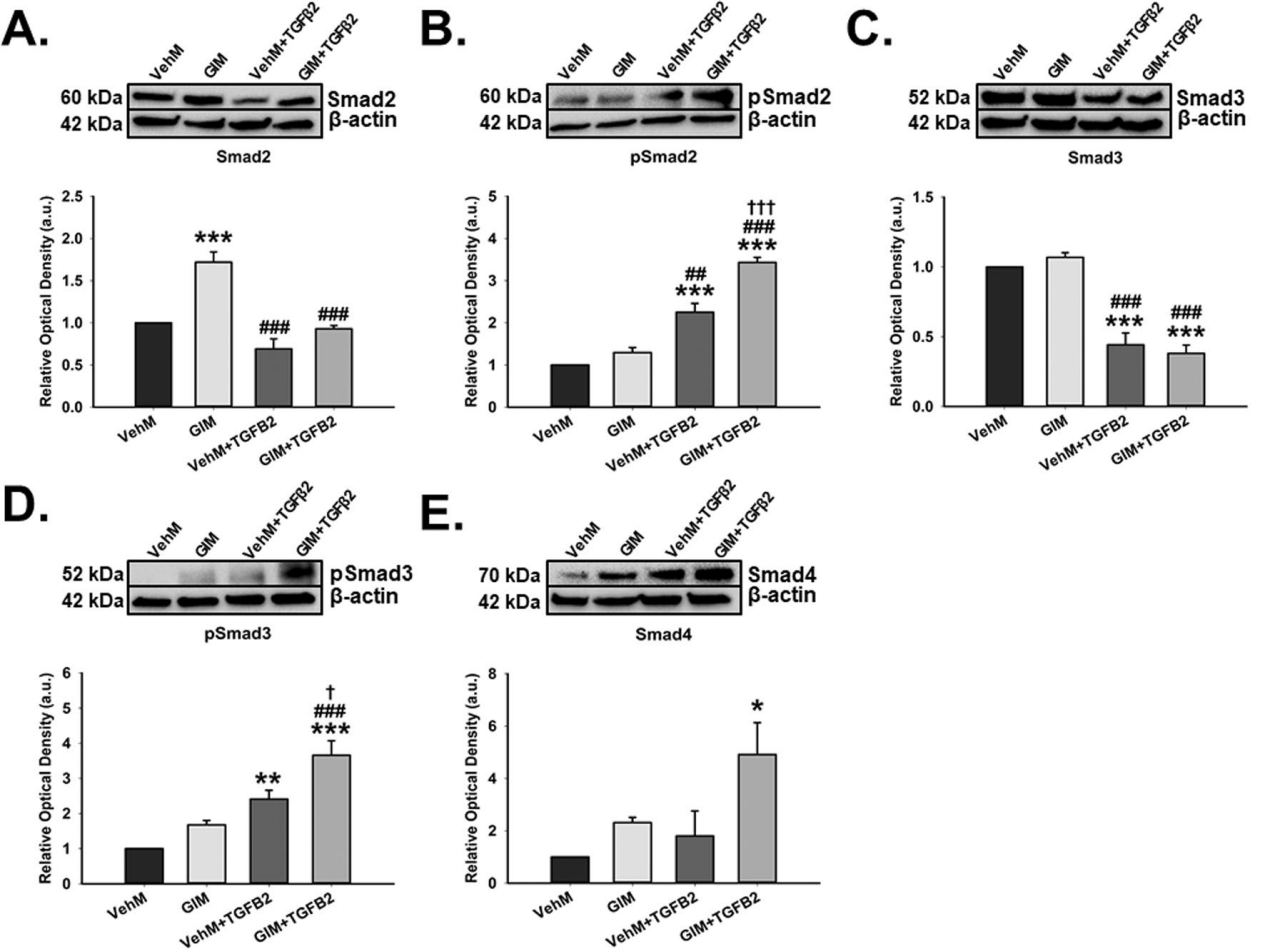
**GIMs activate Smad-dependent TGFβ signaling in the presence of exogenous TGFβ2 in hTM cells.** Primary hTM cells were cultured in the presence or absence of 100 nM dexamethasone for 4 weeks in complete growth media. Cells were subsequently removed using 20 mM ammonium hydroxide solution to obtain GIMs and vehicle control matrices (VehMs). Same strain, fresh primary hTM cells were then seeded on these matrices with or without exogenous 5 ng/mL TGFβ2 in 1% fetal bovine serum growth media for 24 hours. Protein was extracted for Western blot analysis. β-Actin was used as an internal control for normalization. Representative cropped blots (top) and densitometric analysis (bottom) of **(A)** Smad2, **(B)** Phosphorylated Smad2 (pSmad2), **(C)** Smad3, **(D)** Phosphorylated Smad3 (pSmad3), and **(E)** Smad4. *Columns and error bars*; means and standard error of mean (SEM). One-way ANOVA with the Tukey pairwise comparisons post hoc test was used for statistical analysis. (n = 4 biological replicates. *p < 0.05, **p < 0.01, ***p < 0.001 for the group of interest versus control, VehM. ^##^p < 0.01, ^###^p < 0.001 for the group of interest versus GIM. ^†^p < 0.05, ^†††^p < 0.001 for comparison between VehM + TGFβ2 and GIM + TGFβ2). hTM, human trabecular meshwork. As indicated by the black horizontal demarcating lines between respective blot images, all membranes were re-probed for β-Actin as a housekeeping protein. Full length blots can be found in Supplementary Figs. S5A–E and S6A–E, respectively. Densitometric analyses was done using ImageJ 1.8.0_112 software (https://imagej.nih.gov/ij/, 1997–2018).

Next, we extracted proteins from primary hTM cells (from the same donor used to generate matrices) that had been seeded on VehMs and GIMs for 24 hours in 1% serum media in the presence or absence of 5 ng/mL TGFβ2 treatment. Given the relevance of Smad-dependent TGFβ2 pathways in ocular hypertension and glaucoma^29,49,50^, we performed Western blotting to investigate the expression of total and phosphorylated Smad2 and Smad3 molecules. We also probed for the expression of total Smad4. We found that, in the absence of exogenous TGFβ2, GIMs significantly upregulated Smad2 (1.7-fold, p < 0.001) in hTM cells compared with those on vehicle control matrices (VehMs); in the presence of exogenous TGFβ2, no difference in Smad2 expression was observed between VehMs and GIMs (Fig. 1A). Likewise, in the absence of TGFβ2, there were no differences in the expression of the active form of Smad2 (pSmad2) in hTM cells between GIMs and VehMs. In the presence of TGFβ2, however, pSmad2 was significantly overexpressed by VehMs and GIMs (2.3-fold and 3.4-fold, p < 0.001, respectively) compared with no TGFβ2 treatment. Also, activation of pSmad2 was markedly pronounced in GIM + TGFβ2 groups compared with VehM + TGFβ2 group (Fig. 1B). While there were no expressional differences of Smad3 between VehMs and GIMs in the absence of TGFβ2 in hTM cells, in the presence of TGFβ2, VehMs and GIMs markedly downregulated Smad3 (-2.5-fold, p < 0.001, respectively) compared with their respective counterpart in the absence of TGFβ2 (Fig. 1C). No differences were observed, however, in Smad3 expression between VehM and GIM in the presence of TGFβ2. Moreover, in the absence of exogenous TGFβ2, GIMs had no statistically significant effect on the active form of Smad3 (pSmad3) in hTM cells compared with VehMs. Conversely, in the presence of TGFβ2, pSmad3 was significantly increased by VehMs and GIMs (pSmad3; 2.4-fold, p < 0.01 and 3.7-fold, p < 0.001, respectively); the effect was further enhanced in GIMs versus VehMs when TGFβ2 was added (Fig. 1D). Lastly, GIMs markedly overexpressed Smad4 (4.9-fold, p = 0.018) only in the presence of exogenous TGFβ2 (Fig. 1E).

### GIMs potentiated non-Smad TGFβ2 pathways in hTM cells regardless of exogenous TGFβ2

**Figure 2:**
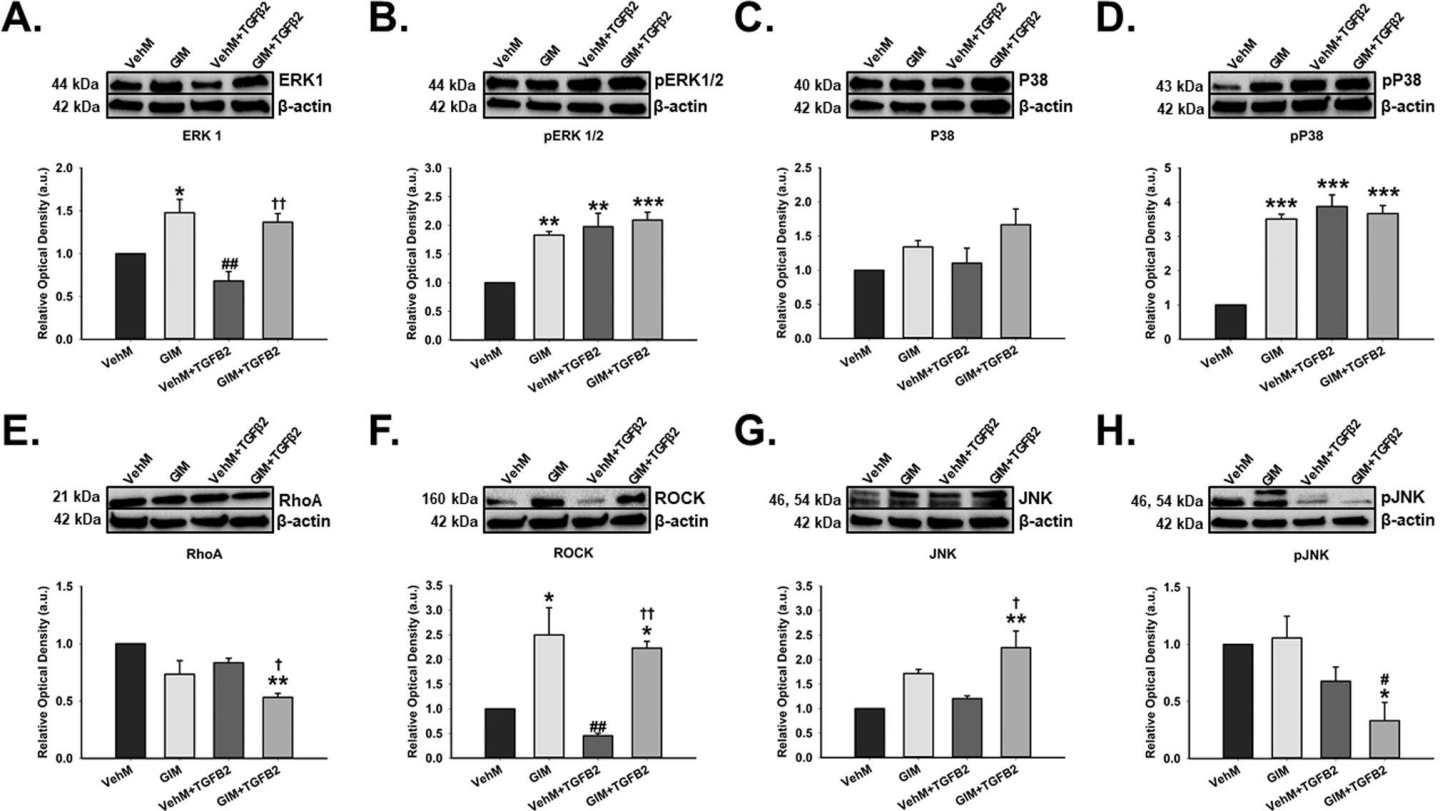
**GIMs potentiate non-Smad-dependent TGFβ signaling with or without exogenous TGFβ2 in hTM cells.** Primary hTM cells were cultured in the presence or absence of 100 nM dexamethasone for 4 weeks in complete growth media. Cells were subsequently removed using 20 mM ammonium hydroxide solution to obtain GIMs and vehicle control matrices (VehMs). Same strain, fresh primary hTM cells were then seeded on these matrices with or without exogenous 5 ng/mL TGFβ2 in 1% fetal bovine serum growth media for 24 hours. Protein was extracted for Western blot analysis. β-Actin was used as an internal control for normalization. Representative cropped blots (top) and densitometric analysis (bottom) of **(A)** Extracellular signal regulated kinase 1 (ERK1), **(B)** Phosphorylated ERK1/2 (pERK1), **(C)** P38, **(D)** Phosphorylated P38 (pP38), **(E)** RhoA, **(F)** Rho-associated protein kinase 1 (ROCK1), **(G)** c-Jun N-terminal kinases (JNK), and **(H)** Phosphorylated JNK (pJNK). *Columns and error bars*; means and standard error of mean (SEM). One-way ANOVA with the Tukey pairwise comparisons post hoc test was used for statistical analysis. (n = 4 biological replicates. *p < 0.05, **p < 0.01, ***p < 0.001 for the group of interest versus control, VehM. ^#^p < 0.05, ^##^p < 0.01 for the group of interest versus GIM. ^†^p < 0.05, ^††^p < 0.01 for VehM + TGFβ2 versus GIM + TGFβ2). hTM, human trabecular meshwork. Black horizontal demarcating lines between the blots indicate two images taken from two different parts of the same membrane blot. Full length blots can be found in Supplementary Figs. S7A–H and S8A–H, respectively. Densitometric analyses was done using ImageJ 1.8.0_112 software (https://imagej.nih.gov/ij/, 1997–2018).

Because TGFβ2 signaling pathways implicated in ocular hypertensive phenotypes go beyond activation of just the Smad-dependent molecules^33,51^, we subsequently determined the expressional levels of the non-Smads. These included the mitogen activated protein kinases (for example, extracellular signal regulated kinase [ERK], P38, and c-Jun N-terminal kinases [JNK]) and Rho-GTPases like RhoA and its kinase, Rho-associated protein kinase (ROCK). We observed that, compared with hTM cells seeded on VehMs, GIMs significantly upregulated ERK1 (1.5-fold, p < 0.05) in the absence of exogenous TGFβ2. In the presence of exogenous TGFβ2, GIM-TGFβ2 interaction significantly increased ERK1 (p < 0.01) in hTM cells compared with that of VehM + TGFβ2 group; no statistical significant differences were observed comparing GIM alone and GIM + TGFβ2 groups, or VehM alone and VehM + TGFβ2 groups (Fig. 2A). In the absence of TGFβ2, GIMs markedly overexpressed phosphorylated ERK1/2 (pERK1/2, 1.8-fold, p < 0.01) in hTM cells compared with VehMs. In the presence of TGFβ2, both VehMs and GIMs significantly increased pERK1/2 (twofold and 2.1-fold, p < 0.001, respectively), although no differences were observed between VehM and GIM groups (Fig. 2B). No significant changes in the expressional levels of P38 was observed comparing VehM and GIM groups with or without TGFβ2 (Fig. 2C). In the absence of TGFβ2, GIMs markedly increased the activated form of P38 (pP38, 3.5-fold, p < 0.001) in hTM cells. In the presence of TGFβ2, VehMs and GIMs significantly overexpressed pP38 (3.9-fold and 3.7-fold, p < 0.001, respectively) in hTM cells, although no differences were observed between VehM and GIM (Fig. 2D). Also, compared with VehMs, GIMs significantly downregulated RhoA (-twofold, p = 0.001) in hTM cells only in the presence of TGFβ2 (Fig. 2E). In contrast, in the absence of TGFβ2, RhoA’s kinase, ROCK, was profoundly overexpressed by GIMs (2.5-fold, p < 0.05) compared with VehMs in hTM cells. Similarly, in the presence of TGFβ2, while VehMs had no significant effect, GIMs markedly increased ROCK (2.2-fold, p < 0.05) in hTM cells (Fig. 2F). Finally, in the absence of TGFβ2, there were no changes in JNK or phosphorylated JNK (pJNK) between VehMs and GIMs in hTM cells, although JNK trended towards an increase despite statistical insignificance; however, in the presence of TGFβ2 GIMs significantly elevated JNK (2.3-fold, p < 0.01) (Fig. 2G), but, downregulated pJNK (-3.3-fold, p < 0.05) (Fig. 2H).

### GIMs overexpressed α-smooth muscle actin in the presence of TGFβ2 in hTM cells

**Figure 3:**
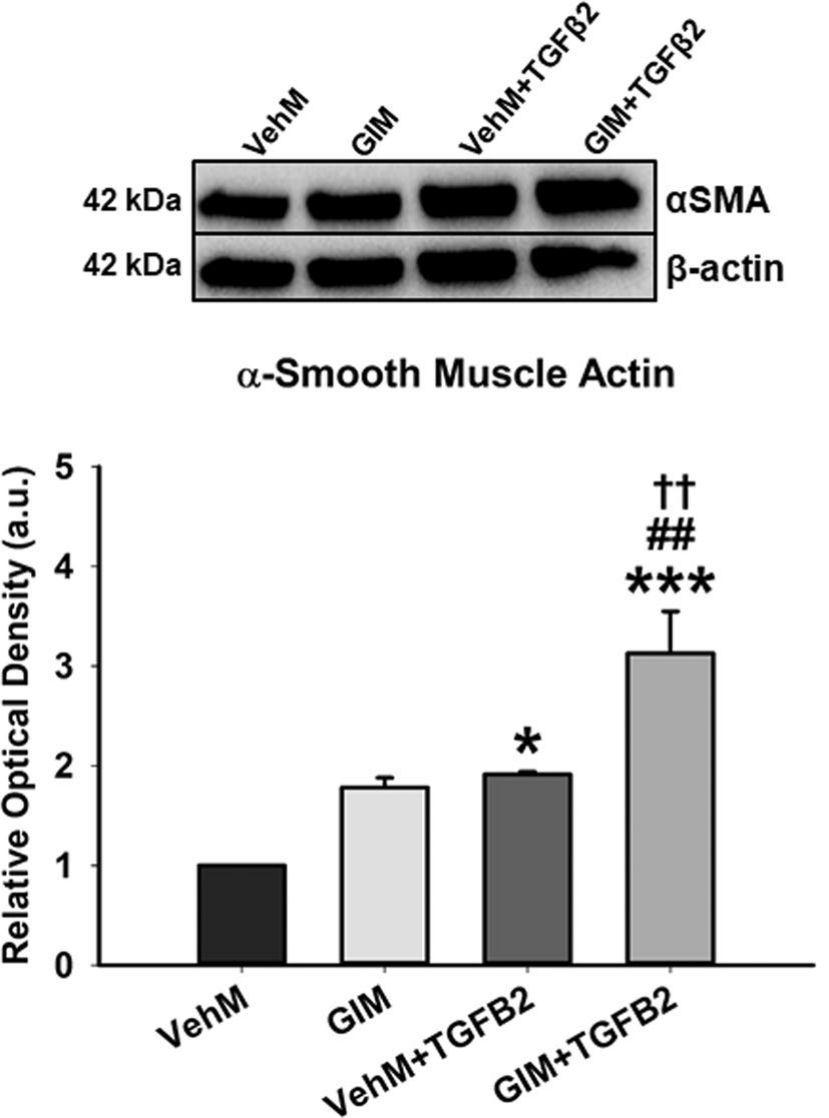
**GIMs increase hTM cell contractility by overexpressing α-smooth muscle actin in the presence of exogenous TGFβ2.** Primary hTM cells were cultured in the presence or absence of 100 nM dexamethasone for 4 weeks in complete growth media. Cells were subsequently removed using 20 mM ammonium hydroxide solution to obtain GIMs and vehicle control matrices (VehMs). Same strain, fresh primary hTM cells were then seeded on these matrices with or without exogenous 5 ng/mL TGFβ2 in 1% fetal bovine serum growth media for 7 days. Protein was extracted for Western blot analysis. β-Actin was used as an internal control for normalization. Representative cropped blot (top) and densitometric analysis (bottom) of α-Smooth muscle actin (αSMA). *Columns and error bars*; means and standard error of mean (SEM). One-way ANOVA with the Tukey pairwise comparisons post hoc test was used for statistical analysis. (n = 4 biological replicates. *p < 0.05, ***p < 0.001 for the group of interest versus control, VehM. ##p < 0.01 for the group of interest versus GIM. ^††^p < 0.01 for comparison between VehM + TGFβ2 and GIM + TGFβ2). hTM, human trabecular meshwork. Black horizontal demarcating line between the blot image signifies two images were taken at different parts of the same membrane blot. Full length blots can be found in Supplementary Figs. S5F and S6F. Densitometric analyses was done using ImageJ 1.8.0_112 software (https://imagej.nih.gov/ij/, 1997–2018).

Given that ocular hypertensive phenotypes induced by TGFβ2 signaling pathways include fibrotic changes in actin cytoskeletal stress fibers^21,33^, we determined whether GIMs could modulate the protein expression of α-smooth muscle actin (αSMA) in seeded hTM cells in the presence or absence of exogenous TGFβ2. We discovered that, compared with hTM cells cultured on VehMs for 7 days, in the absence of exogenous TGFβ2, GIMs trended towards overexpression of αSMA, although statistical significance was not reached. However, interaction between GIMs and exogenous TGFβ2 significantly overexpressed αSMA (3.1-fold, p < 0.001) even beyond that of VehM-TGFβ2 interaction (1.9-fold, p < 0.05) (Fig. 3).

### GIMs induced pathological changes in ECM components in hTM cells in the presence or absence of exogenous TGFβ2

TGFβ2 signaling is well-established in ocular hypertension partly because of its potent regulatory role on TM ECM restructuring^16,26,34,52^. Since it is technically challenging to differentiate between existing ECM proteins from the cell-derived matrices with any induced changes in the same genes/proteins, we primarily focused on mRNA levels.

#### ECM structural genes

**Figure 4:**
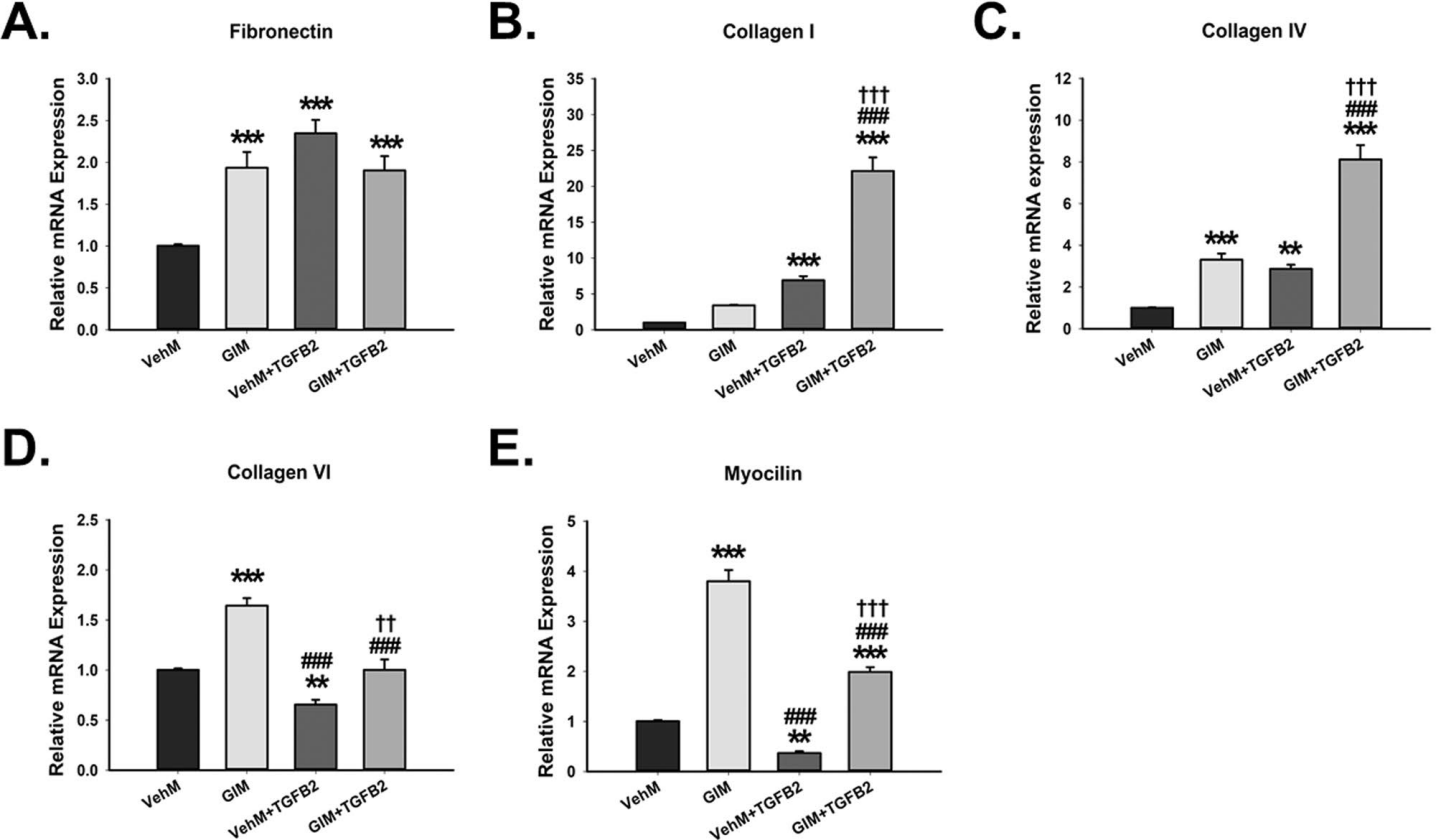
**GIMs increase expression of key ECM structural genes in the presence or absence of exogenous TGFβ2 in hTM cells.** Primary hTM cells were cultured in the presence or absence of 100 nM dexamethasone for 4 weeks in complete growth media. Cells were subsequently removed using 20 mM ammonium hydroxide solution to obtain GIMs and vehicle control matrices (VehMs). Same strain, fresh primary hTM cells were then seeded on these matrices with or without exogenous 5 ng/mL TGFβ2 in 1% fetal bovine serum growth media for 7 days. RNA was extracted for reverse transcription and qPCR. GAPDH was used as an internal control for normalization. Resultant bar graphs for **(A)** Fibronectin, **(B)** Collagen I, **(C)** Collagen IV, **(D)** Collagen VI, and **(E)** Myocilin. *Columns and error bars*; means and standard error of mean (SEM). One-way ANOVA with the Tukey pairwise comparisons post hoc test was used for statistical analysis. (n = 4 biological replicates. **p < 0.01, ***p < 0.001 for the group of interest versus control, VehM. ^###^p < 0.001 for the group of interest versus GIM. ^††^p < 0.01, ^†††^p < 0.001 for VehM + TGFβ2 versus GIM + TGFβ2). hTM, human trabecular meshwork. GAPDH, Glyceraldehyde 3-phosphate dehydrogenase.

First, we determined changes in genes that regulate structural ECM proteins. Compared with hTM cells seeded on control matrices (VehMs) for 7 days, in the absence of TGFβ2, GIMs significantly overexpressed fibronectin (1.9-fold, p < 0.001); similarly, in the presence of TGFβ2, both VehMs and GIMs exhibited markedly increased fibronectin expression (2.3-fold and 1.9-fold, p < 0.001, respectively) (Fig. 4A), although no differences were observed between VehM and GIM groups. Further, in the absence of TGFβ2, compared with VehMs, there were no significant changes in collagen I expression in hTM cells on GIM, although it trended upwards. However, with exogenous TGFβ2, GIMs markedly increased collagen I (22.1-fold, p < 0.001) far more potently than on VehMs (6.9-fold, p < 0.001) (Fig. 4B). In addition, in the absence of TGFβ2, compared with VehMs, GIMs significantly overexpressed collagen IV (3.3-fold, p < 0.001) in hTM cells. Similarly, in the presence of TGFβ2, collagen IV was markedly increased by VehMs and GIMs (2.9-fold, p < 0.01 and 8.1-fold, p < 0.001, respectively), with GIMs showing a more exacerbated response (Fig. 4C). Moreover, in the absence of TGFβ2, GIMs significantly increased collagen VI (1.6-fold, p < 0.001) in hTM cells; however, with exogenous TGFβ2, while VehMs significantly decreased collagen VI (-1.4-fold, p < 0.01), GIM-induced changes were not any different from control VehM (Fig. 4D); although it trended to decrease in comparison with GIM alone without TGFβ2. Finally, in the absence of TGFβ2, compared with VehMs, GIMs significantly increased myocilin (3.8-fold, p < 0.001), which is well-documented as a glucocorticoid response gene, in hTM cells. However, with exogenous TGFβ2, while VehMs markedly suppressed myocilin (-2.5-fold, p < 0.01), GIMs markedly overexpressed it (twofold, p < 0.001) in hTM cells (Fig. 4E); although it trended towards a reduction in comparison with GIM alone without TGFβ2.

#### Matricellular genes

**Figure 5:**
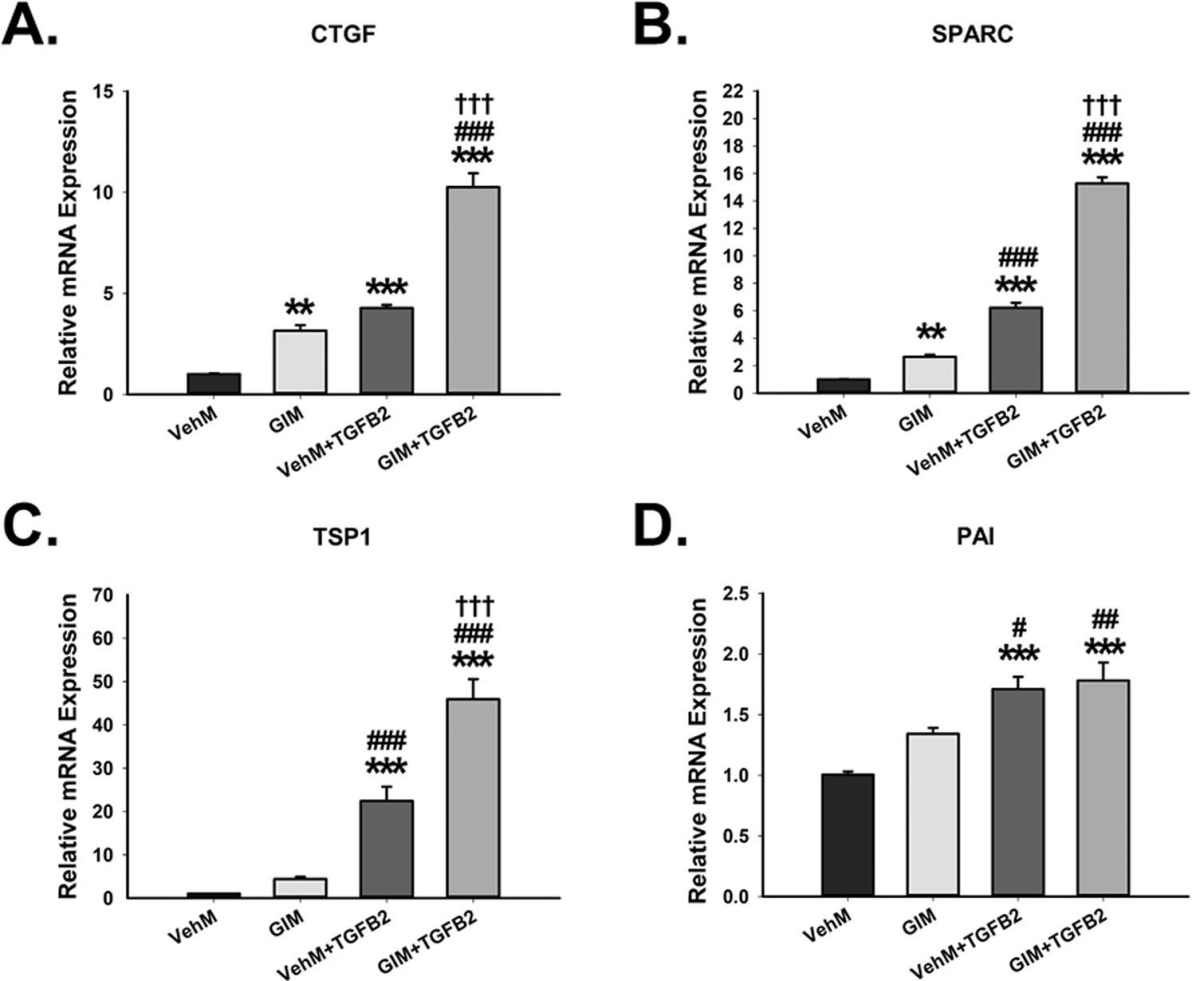
**GIMs overexpress crucial matricellular genes with or without exogenous TGFβ2 in hTM cells.** Primary hTM cells were cultured in the presence or absence of 100 nM dexamethasone for 4 weeks in complete growth media. Cells were subsequently removed using 20 mM ammonium hydroxide solution to obtain GIMs and vehicle control matrices (VehMs). Same strain, fresh primary hTM cells were then seeded on these matrices with or without exogenous 5 ng/mL TGFβ2 in 1% fetal bovine serum growth media for 7 days. RNA was extracted for reverse transcription and qPCR. GAPDH was used as an internal control for normalization. Bar graphs for **(A)** Connective tissue growth factor (CTGF), **(B)** Secreted protein acidic and rich in cysteine (SPARC), **(C)** Thrombospondin 1 (TSP1), and **(D)** Plasminogen activator inhibitor (PAI). *Columns and error bars*; means and standard error of mean (SEM). One-way ANOVA with the Tukey pairwise comparisons post hoc test was used for statistical analysis. (n = 4 biological replicates. **p < 0.01, ***p < 0.001 for the group of interest versus control, VehM. ^#^p < 0.05, ^##^p < 0.01, ^###^p < 0.001 for the group of interest versus GIM. ^†††^p < 0.001, for VehM + TGFβ2 versus GIM + TGFβ2). hTM, human trabecular meshwork. GAPDH, Glyceraldehyde 3-phosphate dehydrogenase.

We next investigated matricellular genes that are involved in ECM reorganization and ECM-cell interaction^1,53^. Without exogenous TGFβ2, compared with hTM cells that had been cultured on VehMs for 7 days, GIMs markedly overexpressed connective tissue growth factor (CTGF; 3.2-fold, p = 0.01), and secreted protein, acidic and rich in cysteine (SPARC; 2.6-fold, p < 0.01); no statistically significant differences were observed for thrombospondin 1 (TSP1) and plasminogen activator inhibitor (PAI), although they trended towards elevation. With exogenous TGFβ2, significantly increased expressions of CTGF (4.3-fold and 10.3-fold, p < 0.001, respectively), SPARC (6.2-fold and 15.3-fold, p < 0.001, respectively), TSP1 (22.4-fold and 45.9-fold, p < 0.001, respectively) and PAI (1.7-fold and 1.8-fold, p < 0.001, respectively) were observed comparing VehM with GIM (Fig. 5A–D). The effect was exaggerated in cells on GIMs treated with TGFβ2 for CTGF, SPARC and TSP1 compared with those on VehMs.

#### ECM crosslinking genes

**Figure 6:**
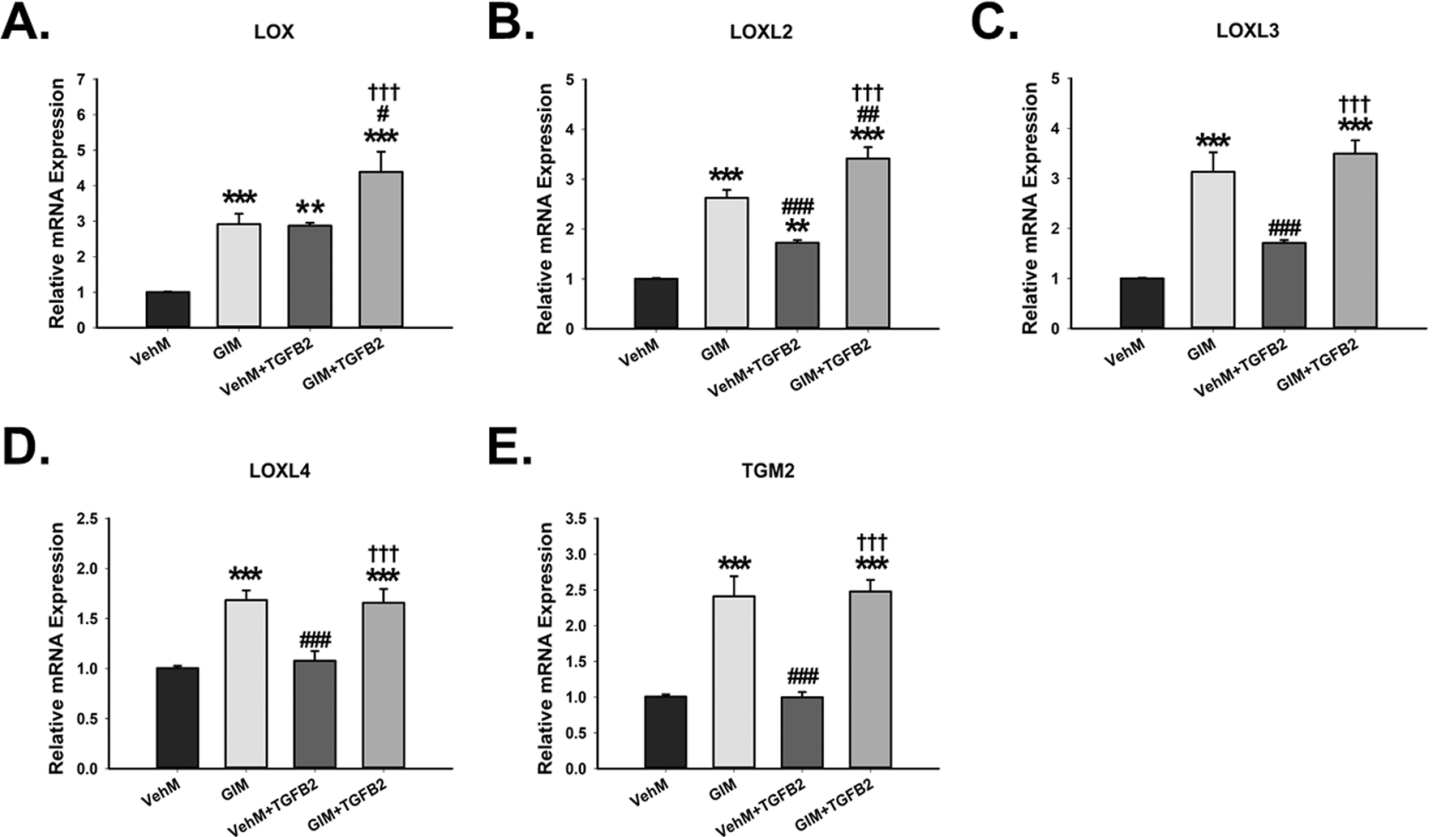
**GIMs upregulate ECM crosslinking genes in the presence or absence of exogenous TGFβ2 in hTM cells.** Primary hTM cells were cultured in the presence or absence of 100 nM dexamethasone for 4 weeks in complete growth media. Cells were subsequently removed using 20 mM ammonium hydroxide solution to obtain GIMs and vehicle control matrices (VehMs). Same strain, fresh primary hTM cells were then seeded on these matrices with or without exogenous 5 ng/ml TGFβ2 in 1% fetal bovine serum growth media for 7 days. RNA was extracted for reverse transcription and qPCR. GAPDH was used as an internal control for normalization. Bar graph for the gene expression of **(A)** Lysyl Oxidase (LOX), **(B)** Lysyl Oxidase-Like 2 (LOXL2), **(C)** Lysyl Oxidase-Like 3 (LOXL3), **(D)** Lysyl Oxidase-Like 4 (LOXL4), and **(E)** Tissue transglutaminase 2 (TGM2). *Columns and error bars*; means and standard error of mean (SEM). One-way ANOVA with the Tukey pairwise comparisons post hoc test was used for statistical analysis. (n = 4 biological replicates. *p < 0.05, **p < 0.01, ***p < 0.001 for the group of interest versus control, VehM. #p < 0.05, ^##^p < 0.01, ^###^p < 0.001 for the group of interest versus GIM. ^†††^p < 0.001 for VehM + TGFβ2 versus GIM + TGFβ2). hTM, human trabecular meshwork. GAPDH, Glyceraldehyde 3-phosphate dehydrogenase.

Enzymes responsible for ECM crosslinking have been implicated in tissue stiffening, glaucoma and TGFβ signaling^10,37,54–56^. Thus, we next determined if pathological matrices modulate these genes. In the absence of TGFβ, compared with hTM cells cultured on VehMs for 7 days, GIMs significantly overexpressed lysyl oxidase (LOX; 2.9-fold, p < 0.001), LOX-like 2 (LOXL2; 2.6-fold, p < 0.001), LOXL3 (3.1-fold, p < 0.001), LOXL4 (1.7-fold, p < 0.001), and tissue transglutaminase (TGM2; 2.4-fold, p < 0.001) (Fig. 6A–E). With exogenous TGFβ2, LOX (2.9-fold, p < 0.01 and 4.4-fold, p < 0.001, respectively) and LOXL2 (1.7-fold, p < 0.01 and 3.4-fold, p < 0.001, respectively) were profoundly increased by VehMs and GIMs; the response was more potent in GIM group versus VehM groups. However, in the presence of TGFβ2, while VehMs had no marked effect on LOXL3, LOXL4 or TGM2, GIMs significantly overexpressed them (LOXL3: 3.5-fold, p < 0.001; LOXL4: 1.7-fold, p < 0.001; and TGM2: 2.5-fold, p < 0.001). Significant differences were thus observed between VehM and GIM groups with exogenous TGFβ2.

#### Genes responsible for ECM turnover

**Figure 7:**
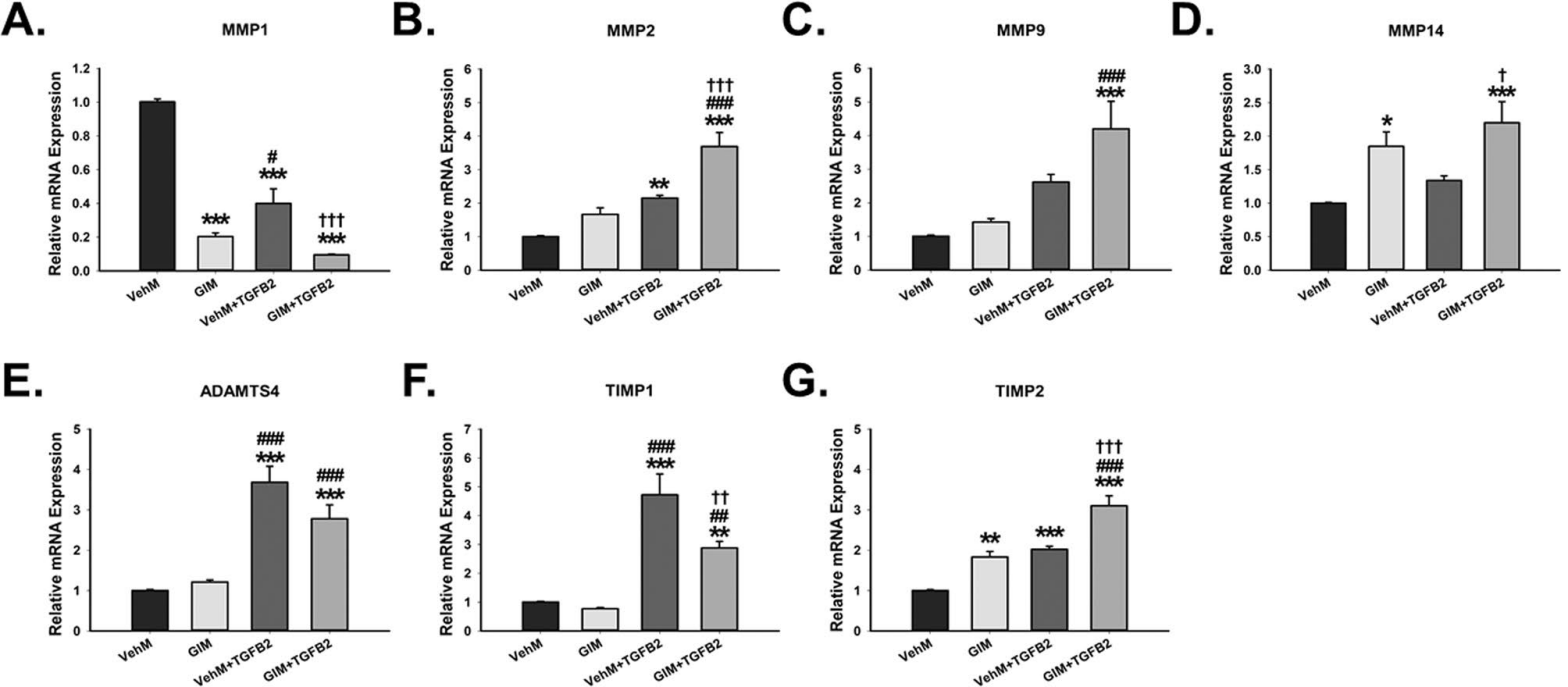
**GIMs modulate specific genes responsible for ECM turnover with or without exogenous TGFβ2 in hTM cells.** Primary hTM cells were cultured in the presence or absence of 100 nM dexamethasone for 4 weeks in complete growth media. Cells were subsequently removed using 20 mM ammonium hydroxide solution to obtain GIMs and vehicle control matrices (VehMs). Same strain, fresh primary hTM cells were then seeded on these matrices with or without exogenous 5 ng/ml TGFβ2 in 1% fetal bovine serum growth media for 7 days. RNA was extracted for reverse transcription and qPCR. GAPDH was used as an internal control for normalization. Bar graph for the gene expression of **(A)** Matrix metalloproteinase 1 (MMP1), **(B)** MMP2, **(C)** MMP9, **(D)** MMP14, **(E)** A disintegrin and metalloproteinase with a thrombospondin motif 4 (ADAMTS4), **(F)** Tissue inhibitor of matrix metalloproteinase 1 (TIMP1), and **(G)** TIMP2. *Columns and error bars*; means and standard error of mean (SEM). One-way ANOVA with the Tukey pairwise comparisons post hoc test was used for statistical analysis. (n = 4 biological replicates. *p < 0.05, **p < 0.01, ***p < 0.001 for the group of interest versus control, VehM. ^#^p < 0.05, ^##^p < 0.01, ^###^p < 0.001 for the group of interest versus GIM. ^†^p < 0.05, ^††^p < 0.01, ^†††^p < 0.001 for VehM + TGFβ2 versus GIM + TGFβ2). hTM, human trabecular meshwork. GAPDH, Glyceraldehyde 3-phosphate dehydrogenase.

To determine whether the GIM-induced overexpression of specific ECM structural genes (Fig. 4), matricellular genes (Fig. 5) and ECM crosslinking genes (Fig. 6) in hTM cells could be physiological or pathological, we probed for the gene expression of specific matrix metalloproteinases (MMPs) implicated in aqueous humor outflow and TM ECM degradation. We observed that, in hTM cells, compared with VehMs, MMP1 was significantly reduced by all experimental groups: GIMs, interaction between VehMs and TGFβ2, and GIM-TGFβ2 interaction (-5-fold, -2.5-fold and -10-fold, p < 0.001, respectively); the greatest reduction was observed with GIM-TGFβ2 interaction (Fig. 7A). However, in the absence of TGFβ2, GIM-induced MMP2 was not any different from VehMs in hTM cells; in the presence of TGFβ2, MMP2 was significantly overexpressed by VehMs and GIMs (2.2-fold, p < 0.01 and 3.7-fold, p < 0.001, respectively), with a greater effect seen in the latter group (Fig. 7B). In addition, compared with VehMs, MMP9 was markedly upregulated in hTM cells only when GIMs interacted with TGFβ2 (4.2-fold, p < 0.001); although statistically insignificant an upward trend was observed with VehM-TGFβ2 interaction compared with control VehM (Fig. 7C). Moreover, without exogenous TGFβ2, compared with VehMs, GIMs significantly increased MMP14 (1.8-fold, p < 0.05) in hTM cells; with exogenous TGFβ2, while VehMs had no significant effect on MMP14, GIMs markedly overexpressed it (2.2-fold, p < 0.001) (Fig. 7D). Moreover, in the absence of TGFβ2, GIMs’ effect on a disintegrin and metalloproteinase with a thrombospondin motif 4 (ADAMTS4) was not any different from VehMs; with exogenous TGFβ2, VehMs and GIMs markedly upregulated ADAMTS4 (3.7-fold and 2.8-fold, p < 0.001, respectively) (Fig. 7E). Next, we determined the expressional levels of tissue inhibitors of matrix metalloproteinases (TIMPs) given their inhibitory role on the function of MMPs in degrading aberrant ECM accumulation. We discovered that, in the absence of exogenous TGFβ2, GIMs had no effect on TIMP1 in hTM cells compared with VehMs. However, in the presence of TGFβ2, both VehMs and GIMs significantly overexpressed TIMP1 (4.7-fold, p < 0.001 and 2.9-fold, p < 0.01, respectively; Fig. 7F), although the effect was greater in VehM than on GIM. Finally, in the absence of exogenous TGFβ2, compared with hTM cells cultured on VehMs, GIMs profoundly increased TIMP2 (1.8-fold, p = 0.001). Similarly, in the presence of TGFβ2, TIMP2 was markedly overexpressed by VehMs and GIMs (two fold and 3.1-fold, p < 0.001, respectively), with a greater effect in cells on GIMs (Fig. 7G).

### Enzymatic activities of MMP2, MMP9 and LOX in conditioned media

**Figure 8:**
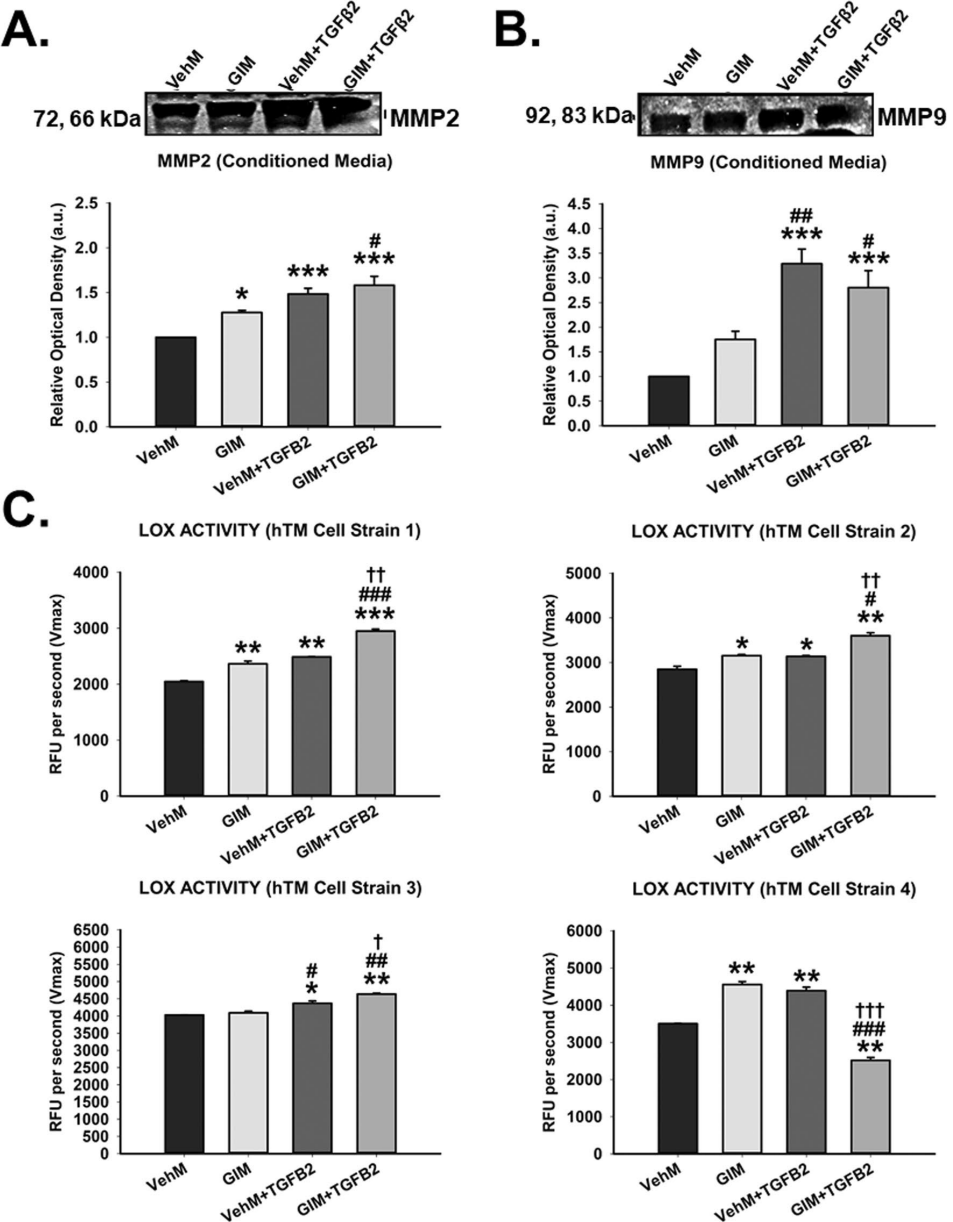
**GIMs increase activities of MMP2 and MMP9 together with LOX in the presence or absence of TGFβ2 in conditioned media.** Conditioned medium from primary hTM cells cultured on vehicle control (VehMs) or GIM substrates for 7 days were collected and normalized to total protein concentration prior to determining enzyme activity in each sample. Gelatin Zymography was used to determine enzyme activity of specific MMPs in the absence or presence of TGFβ2. Representative cropped gel bands of (**A**) MMP2 and (**B**) MMP9 enzymatic activity. Full length gel zymograms are shown in Supplementary Fig. S9. (**C**) Lysyl oxidase enzyme activity was determined as the Vmax (in RFU units per second) for four different cell strains. *Columns and error bars*; means and standard error of mean (SEM). One-way ANOVA with the Tukey pairwise comparisons post hoc test was used for statistical analysis. (n = 4 biological replicates. *p < 0.05, **p < 0.01, ***p < 0.001 for the group of interest versus control, VehM. #p < 0.05, ##p < 0.01, ###p < 0.001 for the group of interest versus GIM. ^†^p < 0.05, ^††^p < 0.01, ^†††^p < 0.001 for VehM + TGFβ2 versus GIM + TGFβ2). hTM, human trabecular meshwork. Densitometric analyses of the zymographs was done using ImageJ 1.8.0_112 software (https://imagej.nih.gov/ij/, 1997–2018). Enzyme activity was measured using the SoftMax Pro 7 Data Acquisition and Analysis Software (Molecular Devices; https://www.moleculardevices.com/products/microplate-readers/acquisition-and-analysis-software/softmax-pro-software).

To investigate whether the upregulation of MMP2, MMP9 and LOX (ECM crosslinking gene) at the gene level translated into functioning proteins, we harvested and subsequently subjected conditioned media from the respective experimental groups to enzymatic assays. As shown in Fig. 8A, in the absence of exogenous TGFβ2, compared with control VehMs, conditioned media from hTM cells seeded on GIMs for 7 days had significantly increased activity of MMP2 (1.3-fold, p < 0.05); similarly, in the presence of TGFβ2, MMP2′ activity in conditioned media from VehMs and GIMs was significantly elevated (1.5-fold and 1.6-fold, p < 0.001, respectively). However, compared with cells on control VehMs in the absence of TGFβ2, conditioned media from hTM cells cultured in the presence of TGFβ2 on VehMs and GIMs had a marked increase in MMP9′ activity (3.3-fold and 2.8-fold, p = 0.001, respectively) (Fig. 8B). Further, we observed that, in the absence of exogenous TGFβ2, conditioned media from three (3) out of the four (4) hTM Cell Strains seeded on GIMs for 7 days had markedly increased LOX activity (Fig. 8C, hTM Cell Strains 1, 2 and 4). Similarly, in the presence of exogenous TGFβ2, significantly elevated LOX activity was found in the conditioned media of three hTM Cells Strains cultured on either VehMs or GIMs (Fig. 8C, hTM Cells Strains 1, 2 and 3), with the latter being more pronounced than the former or GIM alone.

### TGFβRI kinase inhibitor repressed GIM-induced overexpression of CTGF

**Figure 9:**
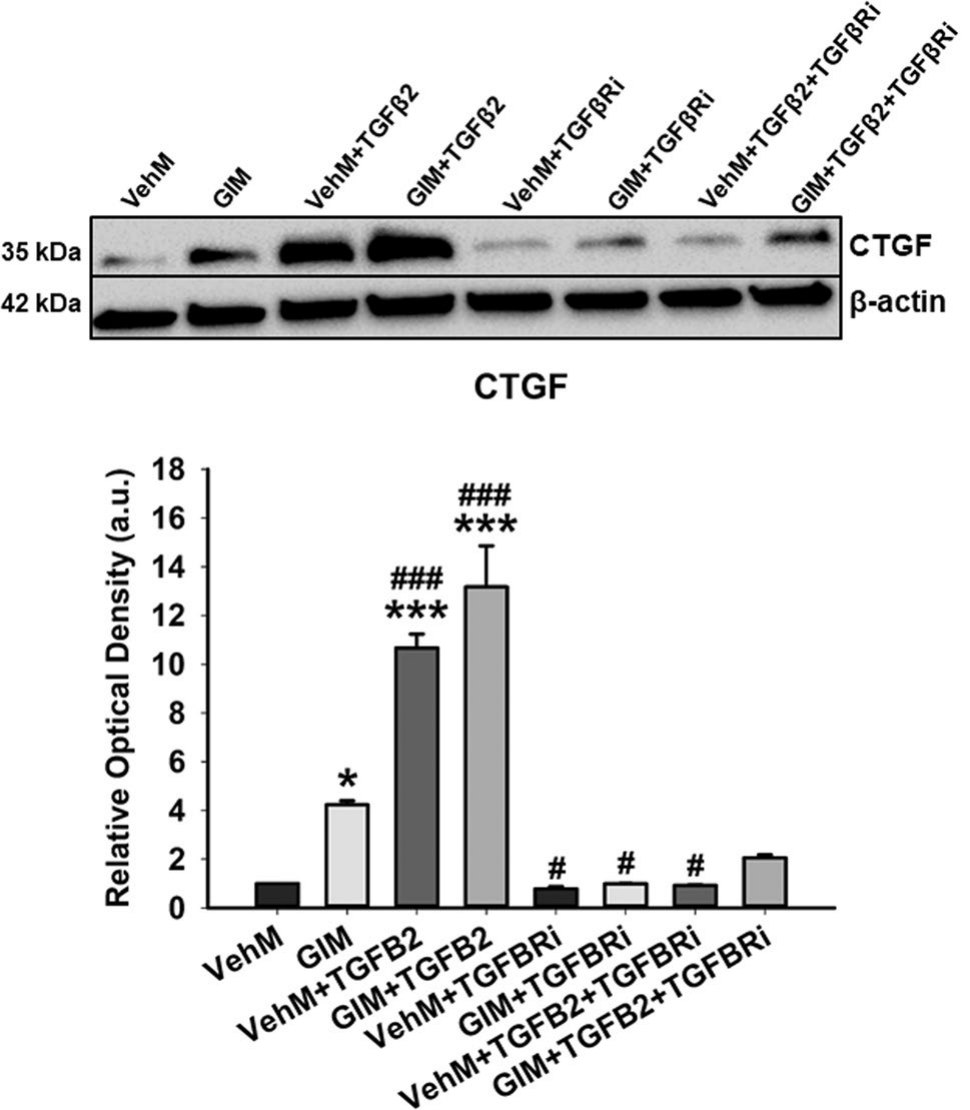
**GIM- and/or TGFB2-induced overexpression of connective tissue growth factor in hTM cells is attenuated by TGFβRI kinase inhibitor.** Primary hTM cells were cultured in the presence or absence of 100 nM dexamethasone for 4 weeks in complete growth media. Cells were subsequently removed using 20 mM ammonium hydroxide solution to obtain GIMs and vehicle control matrices (VehMs). Same strain, fresh primary hTM cells were then seeded on these matrices with or without exogenous 5 ng/ml TGFβ2 and/or 5 µM type I TGFβ receptor (TGFβRI) kinase inhibitor in 1% fetal bovine serum growth media for 24 hours. Protein was extracted for Western blot analysis. β-Actin was used as a housekeeping protein for normalization. Representative cropped blot (top) and densitometric analysis (bottom) of connective tissue growth factor (CTGF). *Columns and error bars*; means and standard error of mean (SEM). One-way ANOVA with the Tukey pairwise comparisons post hoc test was used for statistical analysis. (n = 4 biological replicates. *p < 0.05, ***p < 0.001 for the group of interest versus control, VehM. ^#^p < 0.05, ^###^p < 0.001 for the group of interest versus GIM).TGFβ2, Transforming growth factor β2, TGFβRi, type I TGFβ receptor kinase inhibitor. hTM, human trabecular meshwork. Black horizontal demarcating lines between the blots indicate two images taken from two different parts of the same membrane blot. Full length blots can be found in Supplementary Figs. S5G and S6G. Densitometric analyses was done using ImageJ 1.8.0_112 software (https://imagej.nih.gov/ij/, 1997–2018).

Finally, we investigated whether selective inhibition of type I transforming growth factor beta receptor (TGFβRI) kinase would attenuate GIM-induced overexpression of connective tissue growth factor (CTGF) with or without exogenous TGFβ2. We cultured primary hTM cells on VehMs and GIMs in 1% serum media for 24 hours in the presence or absence of 5 ng/mL TGFβ2 with or without 5 μM TGFβRI kinase inhibitor (LY364947). We then extracted proteins and performed Western blotting to probe for the expression of CTGF. As shown in Fig. 9, in the absence of TGFβRI kinase inhibitor (TGFβRi) or exogenous TGFβ2, compared with control VehMs, GIMs significantly upregulated CTGF (4.2-fold, p < 0.05) in hTM cells. In the presence of TGFβ2 and absence of TGFβRi, both VehMs and GIMs profoundly overexpressed CTGF (10.7-fold and 13.2-fold, p < 0.001, respectively) in hTM cells. Together, this validated CTGF’ gene expression data in Fig. 5A. However, in the presence of TGFβRi, VehM-/GIM- and/or TGFβ2-induced overexpression of CTGF was profoundly abrogated to levels similar to that of control matrices (VehMs). The same observation was true for αSMA (Supplementary Fig. S2).

## Discussion

The extracellular matrix is a three-dimensional framework of structural and matricellular proteins and serves as a reservoir for soluble signaling molecules such as cytokines secreted by resident cells. There is an ever-increasing amount of literature that documents that physico-chemical and biophysical properties of the matrix can profoundly influence cell behavior. While it is well recognized that the ECM is altered in disease, how such a matrix affects cell fate is not known. For example, in glaucoma, a number of studies demonstrate elevated amounts of fibronectin^57–62^ and connective tissue growth factor^63,64^ in the TM. In addition, elevated levels of profibrotic cytokines^27,65–69^ have been reported in the aqueous humor of glaucomatous patients. It is thus inevitable that resident TM cells are continuously subjected to both secreted factors within the tissue and those circulating and passing through the TM. How TM cells respond to such simultaneous pathologic insults has not been evaluated to the best of our knowledge. Therefore, in this study, we report using for the first time an in vitro system, to determine the role that pathologic matrices (for example, GIMs) play in modulating critical signaling pathways (for instance, TGFβ) intrinsically in hTM cells, and under the influence of exogenous profibrotic cytokines (for instance, TGFβ2) added to this culture system. To this effect, we hypothesize that hTM cell responses to GIMs may primarily be driven by: (1) Biochemical composition and topography of GIMs^11^; (2) Biomechanical properties, given that GIMs are approximately fourfold stiffer than VehMs^11,13^; and/or (3) differential presentation of ligands to cells via interactions between biochemical and biomechanical factors from GIMs.

**Figure 10:**
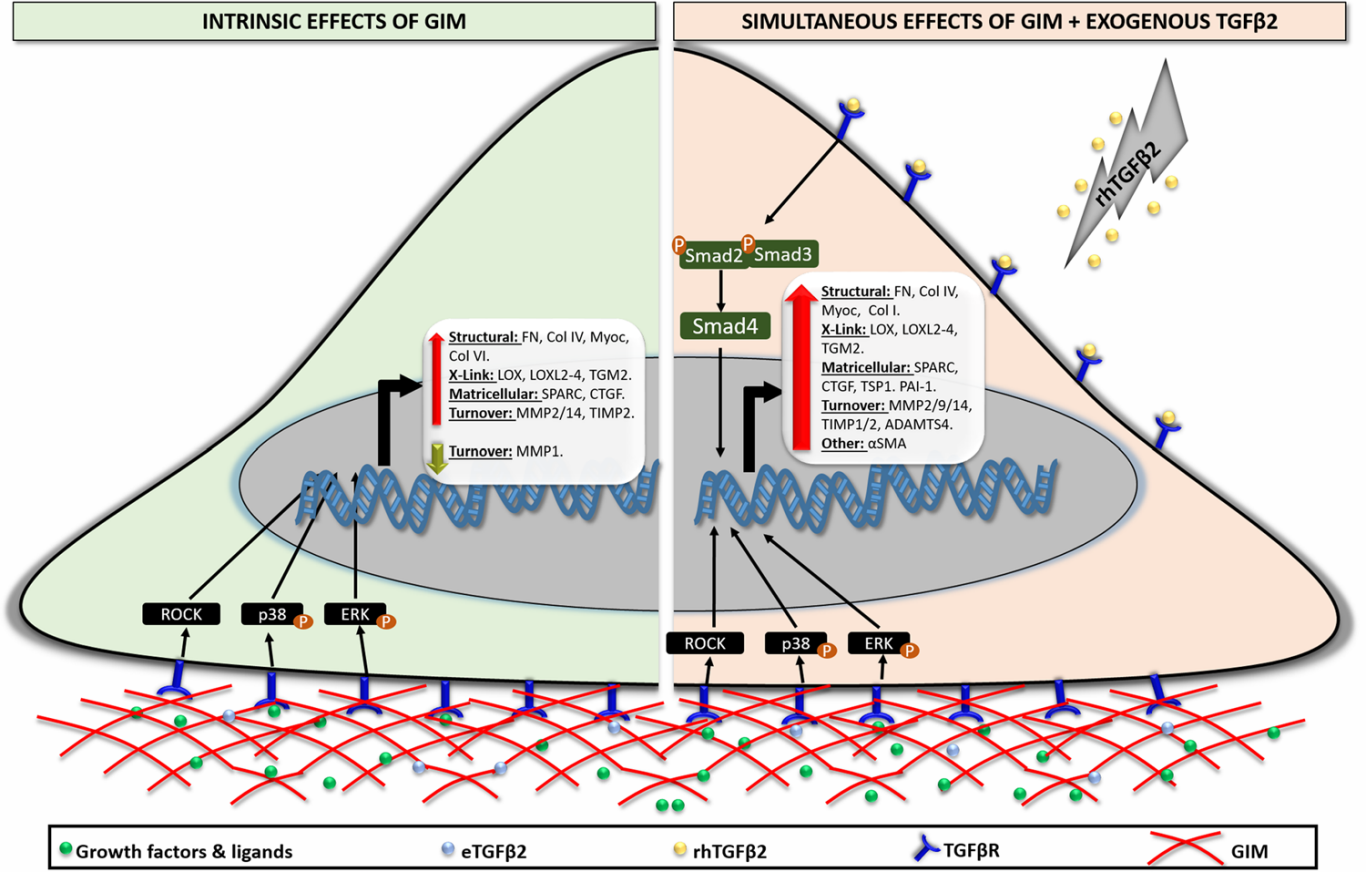
**Schematic representation of the effect of GIMs on TGFβ signaling in hTM cells with or without exogenous TGFβ2.** (**Left cellular compartment**) In the absence of exogenous TGFβ2 or DEX, GIMs activate non-Smad TGFβ2 signaling in correlation with increased expression/activity of specific ECM structural, matricellular, and crosslinking genes/proteins in hTM cells. Imbalances in genes/proteins responsible for ECM turnover (MMPs and TIMP2) lead to impaired ECM degradation, increased ECM deposition and stiffness associated with ocular hypertension. (**Right cellular compartment**) In the presence of exogenous TGFβ2, GIMs potentiate Smad and non-Smad TGFβ2 signaling in association with actin cytoskeletal and more pronounced ECM changes in hTM cells. Concurrent dysregulated ECM turnover (increased MMPs and TIMPs) results in more pronounced ECM accumulation and stiffness implicated in ocular hypertension. *rhTGFβ2* recombinant human transforming growth factor beta 2. *DEX* Dexamethasone. *GIM* glucocorticoid-induced cell-derived matrix. *ECM* Extracellular matrix. *eTGFβ2* ECM-derived TGFβ2. *TGFβR* TGFβ receptor complex. *hTM* Human trabecular meshwork. *MMPs* Matrix metalloproteinases. *TIMPs* Tissue inhibitor of matrix metalloproteinases. *CTGF* Connective tissue growth factor.

Previous studies have determined both in vitro and in vivo that treatment with dexamethasone (DEX) results in overexpression/secretion of active TGFβ2 which may subsequently activate the Smad TGFβ pathway to cause changes in ECM proteins^29^. However, whether ECM remodeling downstream of glucocorticoid receptor signaling is independent of canonical TGFβ signaling is unclear. In addition, it was unknown until now whether the ECM deposited after such DEX treatment further determined cell fate. Thus, first, we showed that, in the absence of any exogenous chemicals (for example, DEX) or growth factors (for instance, TGFβ2), GIMs activated non-Smad-dependent TGFβ signaling to result in additional overexpression of ECM genes/proteins in freshly plated hTM cells or conditioned media (Fig. 10, left cellular compartment). Similar to the interaction between VehMs and exogenous TGFβ2, these aberrant ECM changes encompassed increased accumulation/activity of specific ECM structural, matricellular and crosslinking genes/proteins together with dysregulated ECM turnover. Since there was no exogenous DEX, we infer that the responses directed by GIMs in hTM cells are independent of direct influences from activated glucocorticoid receptor (GR) signaling. Specifically, we showed that, with the exception of phospho-JNK (pJNK), GIMs markedly overexpressed phospho-ERK1/2 (pERK1/2), phospho-P38 (pP38) and Rho-associated protein kinase (ROCK) in hTM cells. These findings were associated with significant increases in ECM structural genes like fibronectin, collagen IV, collagen VI and myocilin; matricellular genes involving connective tissue growth factor (CTGF) and secreted protein, acidic and rich in cysteine (SPARC); ECM crosslinking genes including lysyl oxidase (LOX), LOX-like 2 (LOXL2), LOXL3, LOXL4 and tissue transglutaminase 2 (TGM2); and genes responsible for ECM turnover (matrix metalloproteinase 14 [MMP14] and tissue inhibitor of metalloproteinases 2 [TIMP2]). An imbalance in matrix crosslinking versus degradation may in part play a role in matrix stiffening^70,71^.

Our results are consistent with several studies that have implicated non-Smad TGFβ signaling pathways in aberrant ECM restructuring. For instance, TGFβ2-induced activation of P38 is crucial for the induction of collagen I^72^ and SPARC^73^ in hTM cells. Further, Rho GTPases enhance secretion of bioactive TGFβ2 into the extracellular milieu of hTM cells^74^. Moreover, non-Smads have been implicated in the induction of lysyl oxidases by TGFβ2 in hTM cells^37^. Our group and others have also correlated phospho-ERK1/2 with either the stiffening of DEX-induced hTM cells^11^, or deposition of collagen I/fibronectin in hTM cells cultured on stiffer hydrogels^20^. It was somewhat surprising GIM had no effect on pJNK given that a previous study had implicated pJNK in the induction of all four LOX genes in TGFβ2-stimulated hTM cells^37^. The difference between the findings of the two studies may be due to the differences in the type of glaucomatous insult used: GIMs in ours versus exogenous TGFβ2 in theirs. Additionally, since all the phosphorylated forms of the other non-Smads (pERK1/2, pP38, and ROCK) were upregulated by GIMs in hTM cells, having no GIM-induced change in pJNK may be an efficient mechanism of reducing signaling redundancy.

Concurrent with these changes were GIM-induced increased deposition of ECM structural/matricellular/crosslinking genes. Increased accumulation of fibronectin, collagen IV, collagen VI and myocilin have been implicated in endoplasmic reticulum (ER) stress, ocular hypertension and glaucoma^16,23,43,57,75–77^. Upregulation of fibronectin is particularly critical because not only is its synthesis associated with ER stress^23^, but fundamental to the assembly of other ECM proteins like collagen IV, fibrillin and laminin^78^ which may contribute to altered tissue biomechanics^8^. CTGF and SPARC have also been documented as culprits in ocular hypertension and glaucoma. For example, while CTGF is elevated in pseudoexfoliation glaucoma^63,64,^ and its overexpression causes ocular hypertension in mice^79^, SPARC-null mice have a lower intraocular pressure despite the presence of exogenous TGFβ2^35^. Further, LOX, LOXLs and TGM2 do have their place in glaucoma; single-nucleotide polymorphism in LOXL1 has been implicated in exfoliation glaucoma^55,80^. While all five isoforms of LOX (LOX and LOXL1-4) are upregulated in either gremlin- or TGFβ2-induced hTM cells^37,52^, LOX inhibition increases outflow facility in ex vivo perfusion organ cultures^10^. Similarly, overexpressing TGM2 in mice causes ocular hypertension^56^, and knocking it out reduces TGFβ2-induced ocular hypertension^81^.

Also, there was GIM-stimulated differential modulation of ECM turnover enzymes: MMPs and TIMPs. Because MMP14 and TIMP2 have functional roles in cleaving MMP2 to degrade ECM proteins^82^, one would think GIM-induced overexpression of MMP14 and TIMP2 genes together with increased MMP2 activity in conditioned media would be a homeostatic response to counteract increased ECM synthesis/deposition. However, there is a tipping point in TIMP2′ overexpression beyond which it actually sequesters MMP2 to stall its activity^82^. Additionally, increased activity/expression of MMPs may pathologically cleave and cause dysfunction of key cell surface glycoproteins like prion protein important for maintaining cell-ECM/aqueous homeostasis^83^. Further, the gene expression of other MMPs like MMP1 was significantly downregulated. Moreover, given that crosslinking enzymes covalently crosslink collagen, elastin and fibronectin^84,85^, making them resistant to degradation, GIM-induced increased activity of LOX in conditioned media would hinder MMP2′ activity. Taken together, the combination of GIM-induced activation of non-Smad TGFβ signaling, aberrant ECM synthesis/deposition, increased crosslinking genes/enzymes and dysregulated ECM turnover in hTM cells may contribute to increased stiffness.

Second, we showed that, in the presence of exogenous TGFβ2, GIMs activated both Smad and non-Smad TGFβ signaling in association with changes in actin cytoskeleton, and more pronounced ECM changes in hTM cells compared with either interaction between VehMs and TGFβ2 or GIM alone (Fig. 10, right cellular compartment), in agreement with previous studies^21,33^. This suggests that, synergistic interaction between dual glaucomatous insults (here, being a pathologically remodeled GIM and TGFβ2), as is normally the case in vivo, may further heighten induction of glaucomatous phenotypes/profibrotic conditions beyond what just only one glaucomatous stimulus would do. Notably, GIM-TGFβ2 interaction further increased the expression/activity of ECM genes/proteins that had previously been upregulated by either GIM alone or VehM-TGFβ2 interaction, while overexpressing those that were formerly unaffected by these experimental groups. For instance, besides further upregulating collagen IV in hTM cells beyond that of GIM alone or the interaction between VehMs and TGFβ2, interaction between GIM and TGFβ2 profoundly increased collagen I which was unaffected by GIM alone. Further, this GIM-TGFβ2-induced overexpression of collagen I was significantly beyond that of VehM-TGFβ2 interaction. Similar to the other ECM structural genes^16,77,78,86^, Collagen I has been implicated in glaucoma in association with ER stress, fibrosis and reduced outflow facility^23^. Further, while GIM-TGFβ2 combination heightened the overexpression of CTGF and SPARC in hTM cells beyond GIM alone or VehM-TGFβ2 interaction, other matricellular genes like thrombospondin 1 (TSP1) and plasminogen activator inhibitor (PAI), which were hitherto unchanged by GIM alone, were increased; and this increase was markedly beyond that of VehM-TGFβ2-interaction. GIM-TGFβ2-induced upregulation of TSP1 is important given its elevation in glaucomatous TM^87,88^, and potential contribution to abnormal growth factor signaling^46^. On the other hand, apart from being elevated in the aqueous humor of glaucoma patients^89^, overexpression of PAI suppresses proteolytic degradation of ECM structural proteins by MMPs leading to increased ECM accumulation^90^. Moreover, GIM-TGFβ2 interaction exaggerated the overexpression of LOX and LOXL2 beyond GIM alone or interaction between VehM and TGFβ2, suggesting more pronounced crosslinking of ECM structural proteins. Therefore, although GIM-TGFβ2 combination increased the expression and/or activity of MMP2, MMP9 and/or a disintegrin and metalloproteinase with a thrombospondin motif 4 (ADAMTS4) beyond that of only GIM or VehM-TGFβ2-interaction, repressed MMP1 together with increased LOX-dependent crosslinking of ECM structural proteins would make their proteolytic degradation function challenging. Further, given that both TIMP1 and TIMP2 (genes that inhibit MMP activity) were concurrently upregulated in hTM cells via GIM-TGFβ2 interaction, ECM turnover would most likely be impaired. In aggregate, GIM-TGFβ2 interaction exaggerated the overexpression of target TGFβ molecules in hTM cells beyond GIM alone or interaction between VehM and TGFβ2 most likely because of the heightened synergistic interaction between activated Smad and non-Smad TGFβ signaling molecules. This would probably compound altered biomechanics associated with reduced outflow facility^9,10^ beyond GIM alone or interaction between VehMs and TGFβ2.

Since cells interact with the microenvironment and resident proteins through receptor mediated signaling, we next inhibited type I TGFβ receptor (TGFβRI) kinase using a small molecule inhibitor (LY364947) whose effective concentration (5 μM) has been validated in hTM cells by several studies^29,33,36,37,52^. Importantly, at the concentration used to suppress the kinase activity in vitro, the above-mentioned studies have documented successful inhibition of TGFβ signaling, glucocorticoid-induced endoplasmic reticulum stress, formation of cross-linked actin networks (CLANs), and secretion of major ECM and crosslinking proteins. Consistent with these, here, we observed that, treatment of hTM cells with the same inhibitor abrogated GIM-, VehM-TGFβ2-, or GIM-TGFβ2-induced overexpression of CTGF which may be associated with dysregulated ECM turnover, increased stiffness and elevated intraocular pressure^63,79,91^. We selected CTGF from all the other ECM TGFβ targets because it has been shown to be a subsequent mediator of TGFβ2 signaling^45^. Moreover, overexpressing CTGF in vivo in mice causes ocular hypertension in association with optic nerve damage^79^. Because TGFβ signaling-mediated ocular hypertensive phenotypes go beyond pathological ECM changes to actin cytoskeletal remodeling^21,33^, we also showed that GIM- and/or TGFβ2-induced overexpression of αSMA was attenuated by TGFβRI kinase inhibitor (Supplementary Fig. S2), suggesting inhibition of cell contractility or profibrotic phenotype.

Conversely, a previous study found TGFβ2-induced αSMA was unaffected by inhibitors of Smad3, ERK, P38, ROCK and JNK after 24 hours of respective cotreatment^33^. This difference between our study and theirs may be due to the following reasons. First, we inhibited TGFβRI kinase rather than downstream Smad and non-Smad molecules, suggesting that TGFβ receptor inhibition may be a more effective strategy of ameliorating GIM-induced ocular hypertensive phenotypes. Indeed, in vivo, when used at a concentration of 1% (w/v; 36.7 mM), Kasetti and colleagues^29^ demonstrated such a strategy decreases glucocorticoid-induced ocular hypertension, thus, setting a precedent for this applicability. Future studies will be essential to determine the relative contribution of Smad and non-Smad molecules to GIM- and/or TGFβ2-induced ocular hypertensive phenotypes including altered stiffness in hTM cells. Second, culturing hTM cells on VehMs and GIMs in our study, as opposed to culturing hTM cells on tissue culture plastics/glass coverslips as was done in the previous study likely results in fairly dramatic differences in gene expression levels and cell signaling.

This study is however not without limitations. The effects of VehM-/GIM-induced changes in hTM cell responses were performed on a relatively small yet acceptable sample size (n = 3–5 donors). In compliance with consensus recommendation for TM cell characterization and culture^92^, only hTM cells that responded to DEX treatment with myocilin overexpression were used in our experiments. Thus, determining changes in cells non-responsive to DEX treatment was not done. Further, all our experiments were performed under in vitro conditions. While this is advantageous in delineating the effect of a pathological matrix (for instance, GIMs) on otherwise healthy cells, it does not entirely recapitulate the scenario in vivo where native cells (that may or may not have developed pathology) continue to persist as ECM remodeling occurs. Thus, extrapolations of our data to what is observed in vivo*,* or ex vivo*,* are only suggestive of mechanisms, but should be taken cautiously considering functional changes in outflow resistance and facility were not determined. Clever strategies where anterior segment tissues (healthy and pathologic) are decellularized and subsequently recellularized ex vivo, may be essential to translate our in vitro findings for functional assessments. Developing such models are out of the scope of the current study, although future studies will focus on such methodologies. Also, for the most part, we characterized mRNA expression changes of pathway ECM targets in hTM cells on cell-derived matrices and were therefore, unable to distinguish expression/synthesis of new ECM proteins versus those from the existing matrices. Further studies using specialized techniques such as Stable Isotope Labeling by/with Amino acids in Cell culture (SILAC) may be required to definitively identify target ECM protein changes. Moreover, although DEX was used to simulate the pathological microenvironment, truly glaucomatous cells or matrices were not used in this study. Future studies would be essential to investigate if similar signaling pathways are implicated, although most glaucomatous cells/matrices are hardly free from the effects of previous anti-glaucoma medications that may confound experimental outcomes.

In conclusion, we found that similar to interaction between control VehMs and exogenous TGFβ2, a pathologically remodeled matrix like GIMs, can single-handedly drive pathological changes in hTM cells. These diseased cellular phenotypes are more pronounced upon interaction between this pathologically remodeled matrix and an exogenous glaucomatous stimulus like TGFβ2. Importantly, our results suggest that care must be taken when considering cell-based therapeutic options (for example, stem cell or cell replacement options)^93–96^. This is because similar to resident pathologic cells^97^, a diseased ECM may inadvertently push newly transplanted cells towards a pathologic phenotype rather than ameliorate symptoms especially in the long term. However, inhibiting TGFβRI kinase reverses all these pathological outcomes irrespective of either stimuli or their interaction, suggesting it may potentially be used as an ocular hypotensive target.

## Materials and methods

### Isolation of human trabecular meshwork and subsequent cell culture

Primary human trabecular meshwork (hTM) cells were isolated from donor corneoscleral rims unsuitable for transplant (SavingSight Eye Bank, St. Louis, MO, USA) as described previously^92^. This study is not deemed a Human Subject Research because cells were acquired post-mortem from de-identified donor tissues and is thus considered exempt by University of Houston’s Institutional Review Board (IRB). Nevertheless, all experiments were done in accordance with the tenets of the Declaration of Helsinki. Briefly, TM rings were cut into small pieces after successfully dissecting from corneoscleral rims. These cut pieces were placed with 0.2% (w/v) collagen coated cytodex beads in complete growth medium (Dulbecco’s modified Eagle medium/Nutrient Mixture F-12 [50:50; DMEM/F-12] with 2.5 mM l-glutamine supplemented with 10% fetal bovine serum [FBS], and 1% penicillin/amphotericin [Life Technologies, Carlsbad, CA, USA]). Cells that subsequently moved out of the TM were cultured in complete growth media and generally utilized between passages two (2) and six (6). For characterization of primary hTM cells, all cell strains were examined for dexamethasone (DEX)-induced expression of myocilin as recommended (Supplementary Fig. S3)^98^. Donor details are: 57F, female, age 57 years; 74 M, male, 74 years; 75F, female, age 75 years; and 73 M, male, age 73 years.

### Generation of vehicle control- and glucocorticoid-induced matrices from cultured TM cells

Primary hTM cells were cultured in sterile pretreated 60 mm dishes (5,000–10,000 cells per cm^2^) and amino-silane modified 12 mm glass coverslips^11,99^ in a 24-well culture plate (5000–10,000 cells per cm^2^) using complete growth media. Upon reaching 80–90% confluence, hTM cells were treated with 100 nM dexamethasone (DEX; Sigma-Aldrich Corp., St. Louis, MO, USA) or its vehicle, ethanol (EtOH), for 4 weeks with media changes done on every other day. As previously described, denuding the ECM of its resident cells to obtain vehicle control- (VehMs) or glucocorticoid-induced matrices (GIMs) was done using 20 mM ammonium hydroxide, 0.05% Triton X-100 and deionized water as solvent^11,17^. These cell-derived matrices (CDMs) were then incubated with 50 U/mL DNase I and RNase A for 2 hours, washed thoroughly in Hank’s balanced salt solution (HBSS) prior to subsequent immunocytochemistry (to confirm successful removal of cells from their deposited ECM) and cell culture.

### Biochemical characterization of cell-derived matrices via immunocytochemistry

Appropriate decellularized ECM samples on glass coverslips were fixed with 4% paraformaldehyde in phosphate buffered solution (PBS) at 4 °C for 30 minutes, washed three times, 5 minutes each with PBS; permeabilized with 0.25% Triton X-100 in PBS (pH 7.4) for 10 minutes and washed three times, each for 5 minutes. Subsequently, blocking was done with 5% bovine serum albumin (BSA) in PBS for 30 minutes. Afterwards, samples were incubated overnight at 4 °C with primary antibodies; anti-Fibronectin (catalog number: ab6584, Abcam, Cambridge, MA, USA) and anti-Collagen IV (catalog number: ab6311, Abcam, Cambridge, MA, USA) respectively at 1/250 dilution in 5% BSA/PBS (Primary anti-bodies were omitted as negative controls, data not shown). Following three 5-minute washes in PBS the following day, incubation was done with species-appropriate fluorophore-tagged secondary antibodies (Alexa Fluor 488 Anti-Rabbit and Anti-Mouse; Thermo Fisher Scientific) and CF594-conjugated Phalloidin (catalog number: 00045, Biotium, Fremont, CA, USA) at respective 1/500 dilution at room temperature for 1 hour. After three 5-minute washes, samples were counterstained with 4′6-diamidino-2-phenylindole (DAPI, catalog number: D1306, Fisher Scientific, CA, USA) at 1/10,000 dilution for 5 minutes. After one 5-minute wash, glass coverslips were mounted with mowiol onto slides. Immunofluorescent images were then captured with Zeiss LSM 800 laser scanning confocal microscope (Carl Zeiss, Jena, Germany) or Leica DMi8 inverted fluorescent microscope (Leica Microsystems AG, Germany) with a 20 × objective. For each immunolabelled glass coverslip, 5–10 random locations were imaged. At least 3 glass coverslips were used for each immunolabeling condition for each cell strain with the same imaging settings for cohorts.

### Culturing cells on cell-derived matrices

CDMs obtained were primed with 1% fetal bovine serum (FBS) growth media at room temperature for approximately three (3) to four (4) hours before aspirating the growth media out and culturing cells freshly on them. Primary hTM cells from the same donor (used to derive CDMs) were seeded on CDMs at early passages (5000–10,000 per cm^2^) in 1% FBS growth media (to minimize confounding effects of endogenous growth factors that may be present in serum) with or without 5 ng/mL TGFβ2 (R&D Systems, Minneapolis, MN, USA) treatment for either 24 hours (generally for protein expression of signaling molecules) or 7 days (generally for protein/gene expression of target molecules). A subset of these cultures were probed for inhibition of TGFβ2 signaling with selective type I TGFβ receptor (TGFβRI) kinase inhibitor (LY364947, 5 µM^29,33^, Catalog number: 2718, R&D systems, Minneapolis, MN, USA) for 24 hours. This inhibitor (i.e. LY364947) has been demonstrated to be very efficacious at a concentration of 5 μM in hTM cells by several studies^29,33,36,37,52^. Subsequently, cells were lysed for Western blotting and reverse transcriptase-quantitative polymerase chain reaction (RT-qPCR); their respective conditioned growth media were concurrently harvested and subjected to enzymatic assays to probe activities of specific matrix metalloproteinases (for example, MMP2 and MMP9) and crosslinking enzymes (for instance, lysyl oxidase) (Supplementary Fig. S4).

### Protein isolation and Western blotting

Primary hTM cells cultured on VehMs and GIMs for either 24 hours or 7 days in the presence or absence of TGFβ2 with or without TGFβRI kinase inhibitor were lysed and scraped into radioimmunoprecipitation assay (RIPA) buffer (ThermoScientific, Waltham, MA, USA) supplemented with protease and phosphatase inhibitors (Fisher Scientific, Hampton, NH, USA) on ice, and then subsequently centrifuged at 12,000 g for 15 minutes at 4 °C to pellet and get rid of cellular debris. Supernatants were transferred to fresh tubes and quantified via a modified Lowry assay (DC assay; Biorad, Hercules, CA, USA) with BSA as the standard. Protein lysates were subsequently denatured in a 1:10 mixture of 2-mercaptoethanol and 4 × Laemmli buffer by boiling at 100 °C for 5 minutes. After quickly centrifuging proteins at 15,000×*g* for 30 seconds, equal amount of protein was loaded per well (20 µg) for each sample and ran on denaturing 4–15% gradient polyacrylamide ready-made gels (Biorad); subsequently transferred onto polyvinylidene difluoride (PVDF) membranes by electrophoresis. Membrane blots were blocked in 5% BSA in 1 × tris buffered saline/tween-20 (TBST) for 1 hour. Immunoblots were incubated overnight at 4 °C with specific primary antibodies (Supplementary Table S1) on a rotating shaker. The membrane blot was washed thrice with TBST; each wash lasting for approximately 10 minutes. Subsequent incubation with corresponding HRP-conjugated species-specific secondary antibodies (Supplementary Table S1) for 45 minutes was done, followed by three 10-minute washes with TBST. The protein bands were then visualized using ECL detection reagents (SuperSignal West Femto Maximum Sensitivity Substrate; Life technologies, Grand Island, NY, USA) and imaged with a Bio-Rad ChemiDoc MP imaging system. Respective membrane blots were stripped and probed with β-Actin as a loading control. Data were exported into ImageJ 1.8.0_112 software (https://imagej.nih.gov/ij/, 1997–2018) for densitometric analysis.

### RNA isolation and quantitative real-time PCR

Total RNA was isolated from cells that had been seeded on VehMs and GIMs for 7 days with or without TGFβ2 treatment using an RNA purification kit (Catalog number: 12183025; PureLink RNA Mini kit, Invitrogen, Carlsbad, CA). First-strand cDNA was synthesized using 1 µg of total RNA and the High-Capacity cDNA Reverse Transcription Kit (Catalog number: 4368813; Applied Biosystems, Foster City, CA) following the manufacturer's instructions. Quantitative real-time polymerase chain reaction (qPCR) was performed on 20 ng of the cDNA with specific primers (Supplementary Table S2) and the PowerUp SYBR Green Master Mix kit (Catalog number: A25918; Applied Biosystems, Foster City, CA) in total volumes of 10 µL per reaction using a CFX Connect Real-time System from Bio-Rad Laboratories (Bio-Rad, Hercules, CA, USA). The cycle threshold (Ct) values were obtained from the qPCR equipment and analyzed using the 2^−ΔΔCt^ method with Glyceraldehyde 3-phosphate dehydrogenase (GAPDH) as the housekeeping gene.

### Enzymatic activity assay for specific matrix metalloproteinases in conditioned media

MMP enzyme activity was measured using gelatin zymography as per manufacturer’s instructions. Briefly, primary hTM cells were cultured on VehMs and GIMs with or without TGFβ2 in 1% FBS growth media for 7 days. Conditioned medium was collected, and total protein was quantitated using the Pierce BCA Protein Assay (Catalog number: 23225, ThermoFisher Scientific, Waltham, MA, USA) according to the manufacturer’s instructions. Total protein was normalized per sample prior to loading onto 10% Gelatin Zymogram Gels (15 µg/lane) (Catalog number: ZY00100, ThermoFisher Scientific, Waltham, MA, USA). Gels were washed twice in Zymogram Renaturing Buffer (Catalog number: LC2670, ThermoFisher Scientific, Waltham, MA, USA) at room temperature, prior to incubating in Zymogram Developing Buffer (Catalog number: LC2671, ThermoFisher Scientific, Waltham, MA, USA) overnight with shaking at 37ºC. Gels were then washed and stained before imaging using the Odyssey imaging system (Licor Biosciences, Lincoln, NE, USA). Gel bands were exported into ImageJ software for densitometric analysis.

### Activity assay for crosslinking enzymes in conditioned media

Lysyl oxidase enzyme activity was measured using the Fluorometric Lysyl Oxidase Activity Assay Kit (Catalog number: K928-100, BioVision, Milpitas, CA) according to the manufacturer’s instructions. Briefly, conditioned medium was collected from cultured cells that had been seeded on CDMs for 7 days in 1% serum media in the presence or absence of TGFβ2; and subsequently concentrated tenfold using 10 K MWCO PES Pierce Spin Concentrators (Catalog number: 88512, ThermoFisher Scientific, Waltham, MA). Samples were then normalized to total protein concentration, as described above. Samples were prepared with reaction components and fluorescence was measured (Ex/Em = 535/587) in kinetic mode every 30 seconds for 60 minutes at 37ºC in a black plate using the SpectraMax iD3 Multi-Mode MicroPlate Reader (Molecular Devices, San Jose, CA). The LOX enzyme activity was measured as the Vmax (in RFU units per second) using the SoftMax Pro 7 Data Acquisition and Analysis Software (Molecular Devices; https://www.moleculardevices.com/products/microplate-readers/acquisition-and-analysis-software/softmax-pro-software).

### Statistics

One-way ANOVA followed by Tukey multiple comparisons post hoc test was used for analysis, with *P* values less than 0.05 considered to be statistically significant. Group-wise statistical comparisons are indicated within each figure legend. All data are presented as mean ± SEM, mostly in bar graphs, representative immunofluorescent micrographs, and blots where applicable.

## Supplementary information

Supplementary information.
