# Supplementary material for: Glucocorticoid-induced cell-derived matrix modulates transforming growth factor β2 signaling in human trabecular meshwork cells
Source: Sci Rep. 2020 Sep 24;10:15641. doi: 10.1038/s41598-020-72779-w (PMC7518434; doi:10.1038/s41598-020-72779-w)
Supplement: Supplementary file 1 — Supplementary information. [file 41598_2020_72779_MOESM1_ESM.docx]

**Supplementary Information**

**Glucocorticoid-induced cell-derived matrix modulates transforming growth factor β2 signaling in human trabecular meshwork cells**

Felix Yemanyi^1^, Janice Vranka^2^, VijayKrishna Raghunathan^1,3*^

^1^Department of Basic Sciences, College of Optometry, University of Houston, Houston, TX

^2^Casey Eye Institute, Oregon Health and Science University, Portland, OR

^3^Department of Biomedical Engineering, Cullen College of Engineering, University of Houston, Houston, TX

*Correspondence should be sent to: [vraghunathan@uh.edu](mailto:vraghunathan@uh.edu)


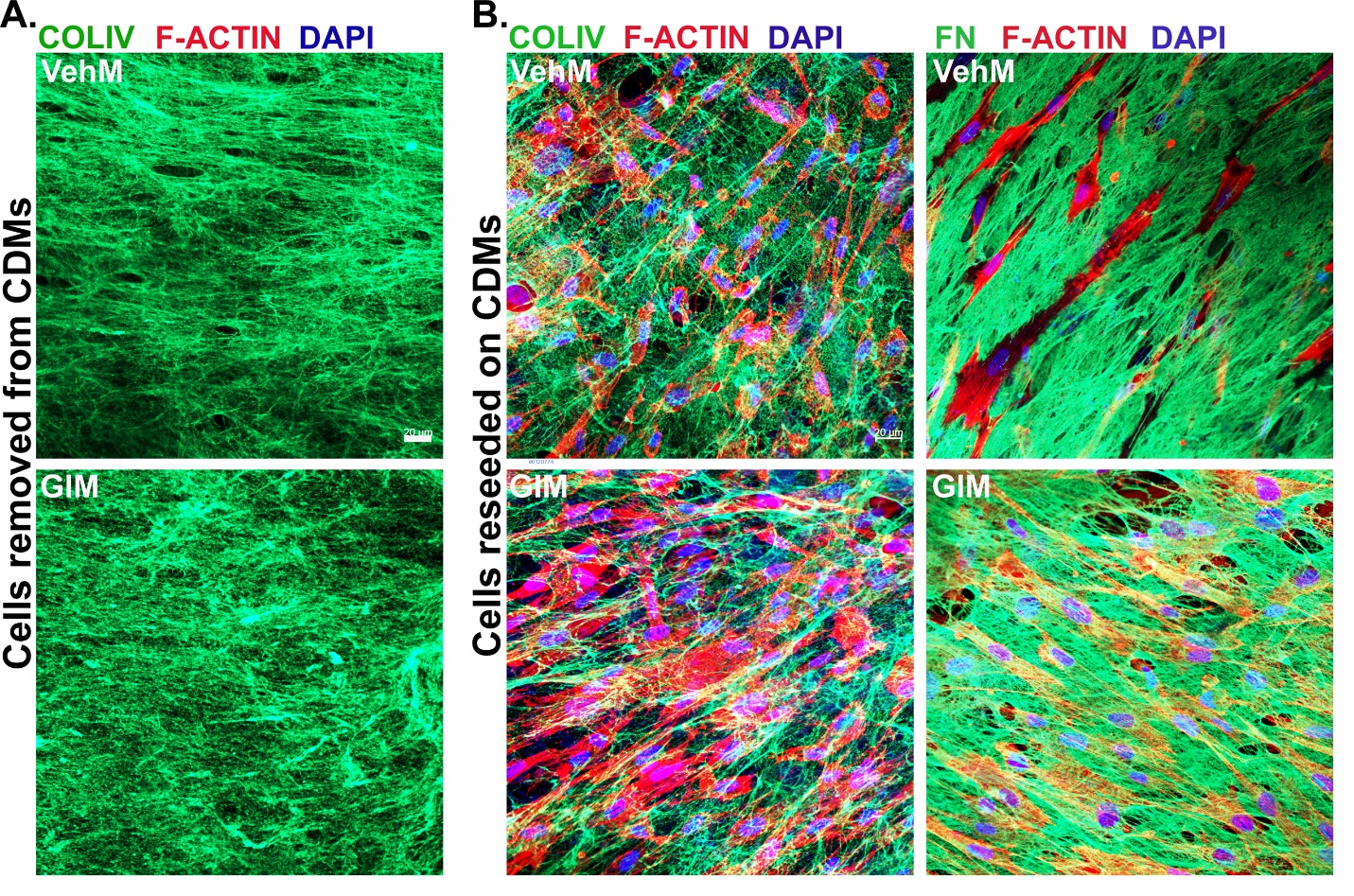
**Supplementary Figure S1. Representative immunolabeled micrographs showing decellularized CDMs and recellularized CDMs. (A)** Decellularized vehicle control- (VehM) and glucocorticoid-induced CDMs (GIM) immunolabelled for collagen IV (COLIV). **(B)** Recellularized VehMs and GIMs immunolabeled for collagen IV (COLIV) and fibronectin (FN) respectively. CDMs, Cell-derived matrices. F-actin, Filamentous actin. DAPI, 4′,6-diamidino-2-phenylindole. Scale bar, 20 µm.



 **Supplementary Figure S2. GIM- and/or TGFB2-induced overexpression of α-smooth muscle actin in hTM cells is abrogated by TGFβRI kinase inhibitor.** Primary hTM cells were cultured in the presence or absence of 100 nM dexamethasone for 4 weeks in complete growth media. Cells were subsequently removed using 20 mM ammonium hydroxide solution to obtain GIMs and vehicle control matrices (VehMs). Same strain, fresh primary hTM cells were then seeded on these matrices with or without exogenous 5 ng/ml TGFβ2 and/or 5 µM type I TGFβ receptor (TGFβRI) kinase inhibitor in 1% fetal bovine serum growth media for 24 hours. Protein was extracted for Western blot analysis. β-Actin was used as a housekeeping protein for normalization. Representative cropped blot of α-smooth muscle actin (αSMA) (n=2 biological replicates). TGFβ2, Transforming growth factor β2. TGFβRi, Type I TGFβ receptor kinase inhibitor. hTM, human trabecular meshwork.





**Supplementary Figure S3. Validation of TM cell strain.** Primary hTM cells, from 4 donors, were cultured in the presence or absence of 100 nM dexamethasone (Dex) for 3 days in complete growth media, after which RNA was isolated and expression of myocilin was in technical triplicate determined by qRT-PCR. Results are mean ± standard deviation, ***p<0.001 compared with vehicle control (EtOH) by paired t-test.


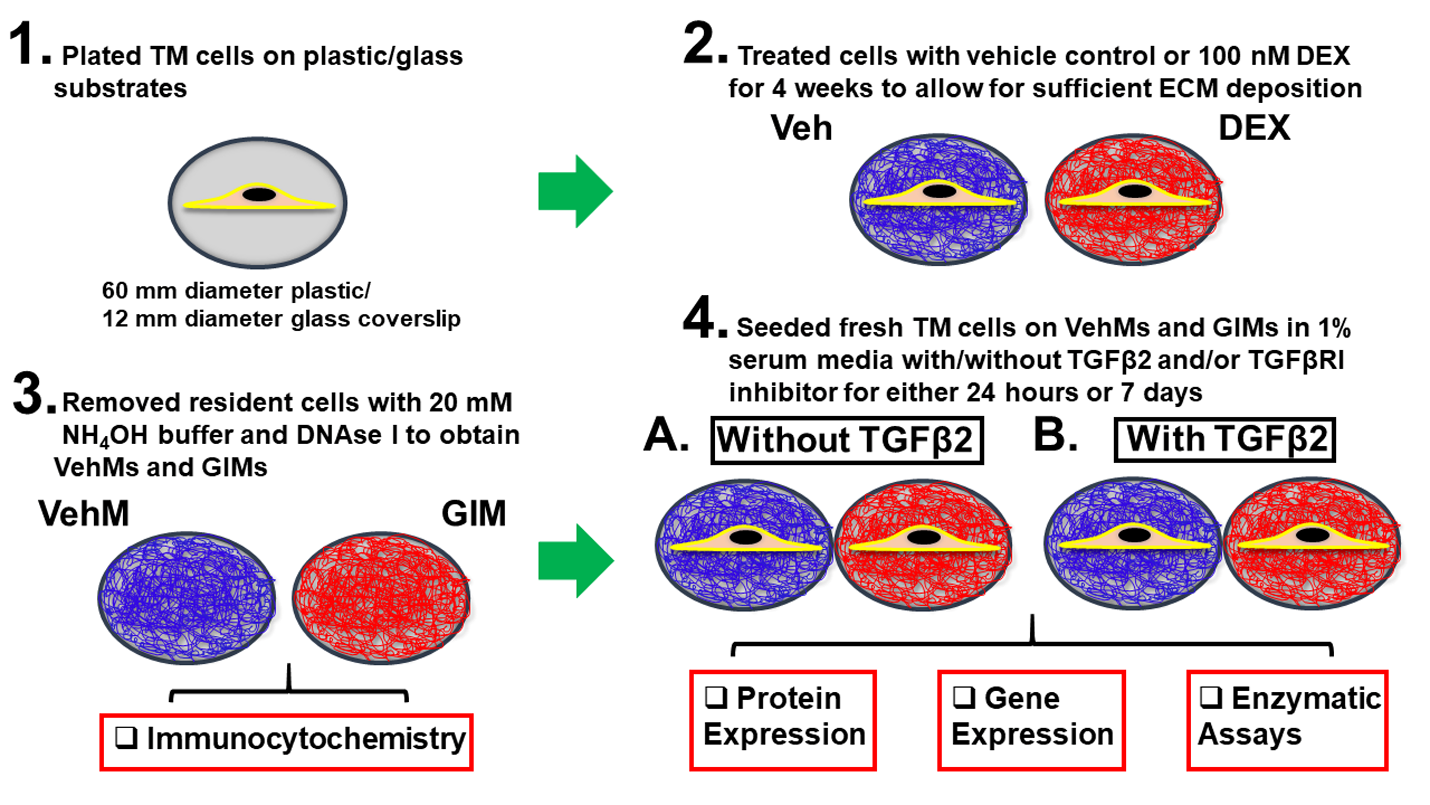


**Supplementary Figure S4. Schematic representation of methods.** TM, Trabecular meshwork. Veh, Vehicle control. DEX, Dexamethasone. ECM, Extracellular matrix. VehM, Vehicle control-induced cell-derived matrix. GIM, Glucocorticoid-induced cell-derived matrix. TGFβ2, Transforming growth factor β2. TGFβRI, Type I transforming growth factor receptor.


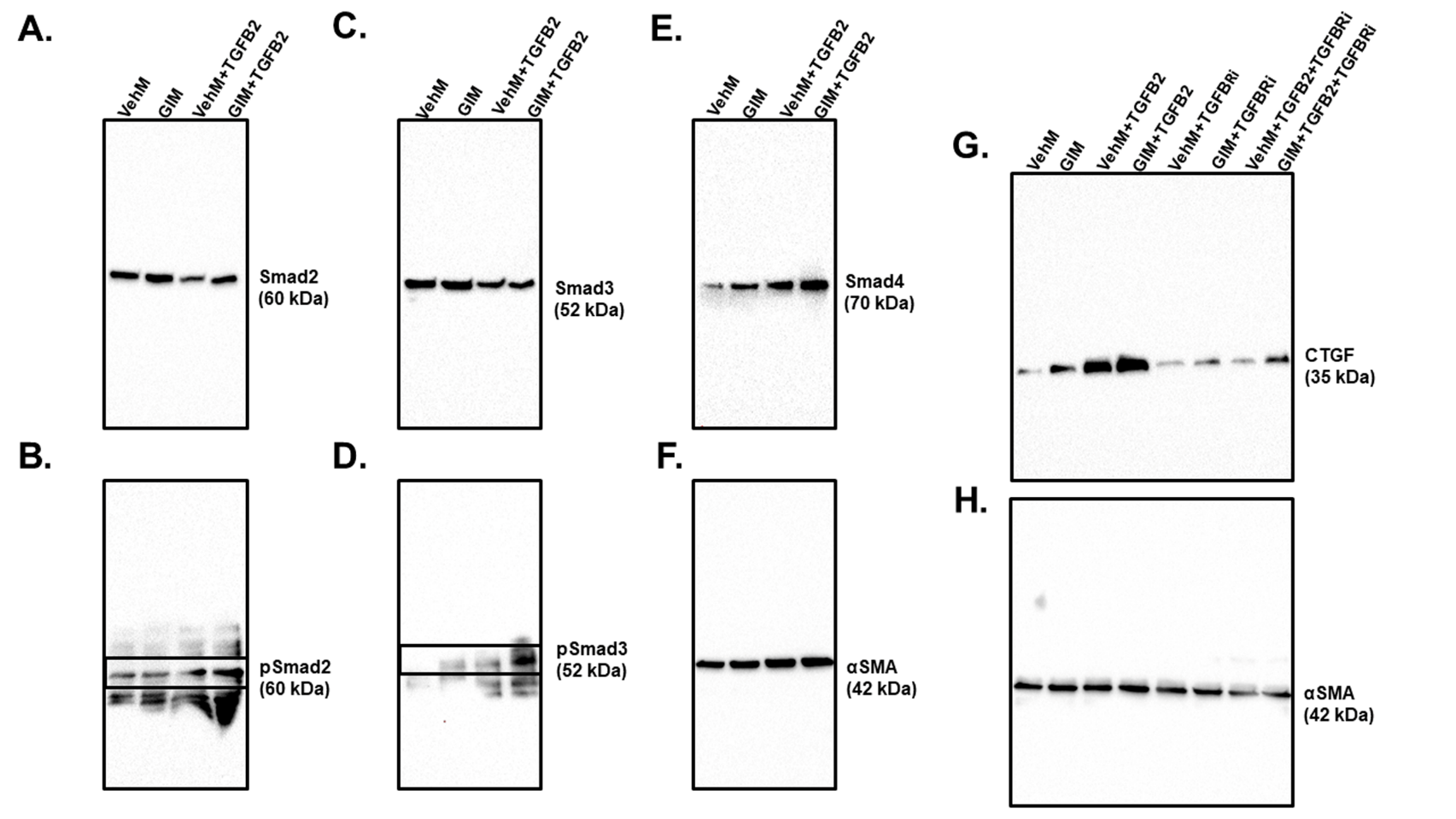


**Supplementary Figure S5. Full length Western Blots of (A)** Smad2 **(B)** pSmad2 **(C)** Smad3 **(D)** pSmad3 **(E)** Smad4 **(F)** αSMA **(G)** CTGF and **(H)** αSMA whose cropped samples are shown in Figure 1A, B, C, D, E; Figure 3, Figure 9 and Supplementary Figure 2 respectively**.**





**Supplementary Figure S6. Full length Western Blots of housekeeping protein β-actin respectively for (A)** Smad2 **(B)** pSmad2 **(C)** Smad3 **(D)** pSmad3 **(E)** Smad4 **(F)** αSMA **(G)** CTGF and **(H)** αSMA in ***Supplementary Figure S5*** whose cropped samples are shown in Figure 1A, B, C, D, E; Figure 3, Figure 9 and Supplementary Figure 2 respectively**.**


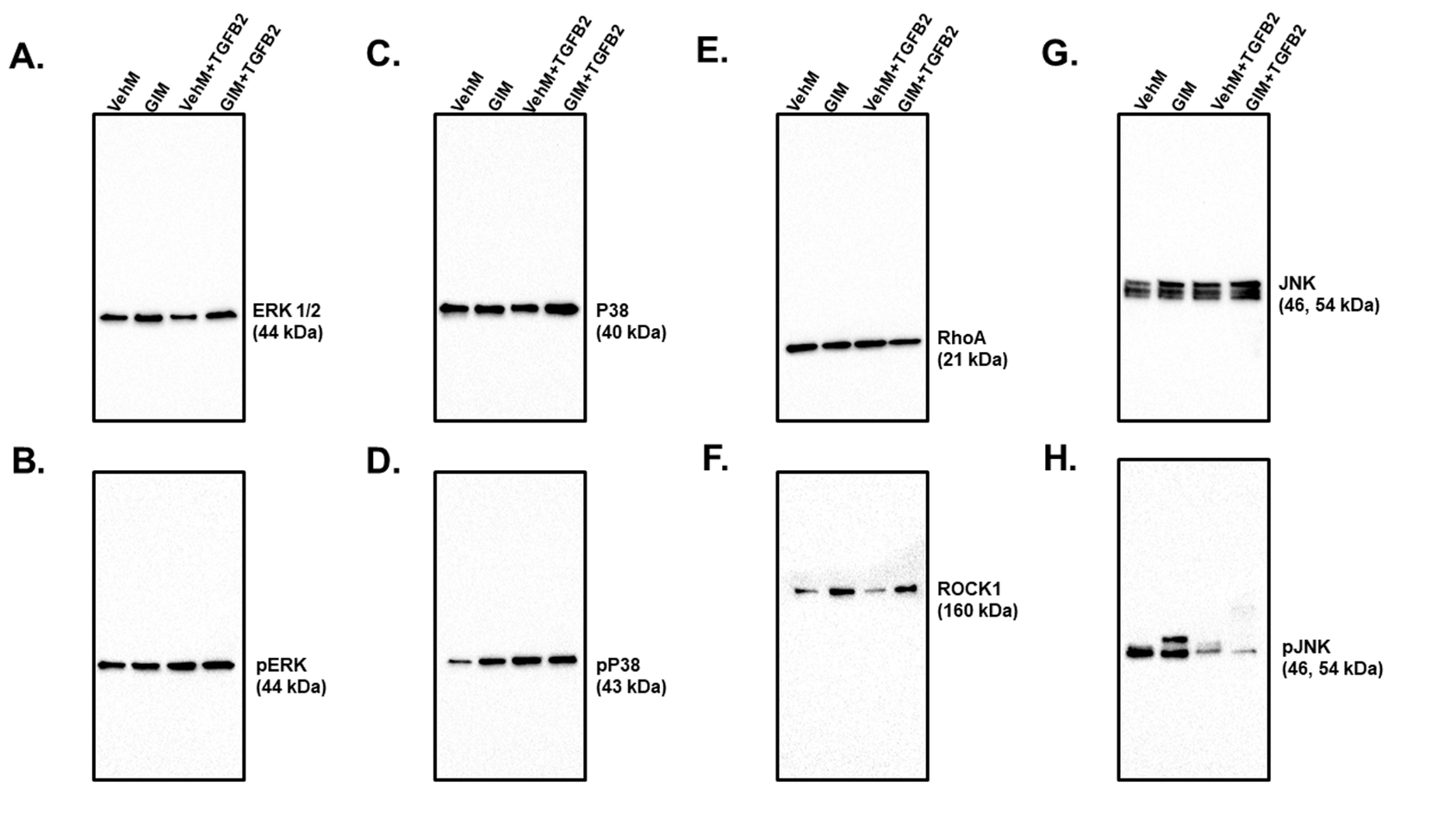
**Supplementary Figure S7. Full length Western Blots of (A)** ERK 1**(B)** pERK 1/2 **(C)** P38 **(D)** Pp38 **(E)** RhoA **(F)** ROCK1 **(G)** JNK and **(H)** pJNK whose cropped samples are shown in Figure 2A, B, C, D, E and H respectively.





**Supplementary Figure S8. Full length Western Blots of housekeeping protein β-actin respectively for (A)** ERK 1**(B)** pERK 1/2 **(C)** P38 **(D)** Pp38 **(E)** RhoA **(F)** ROCK1 **(G)** JNK and **(H)** pJNK in ***Supplementary Figure S7*** whose cropped samples are shown in Figure 2A, B, C, D, E and H respectively.


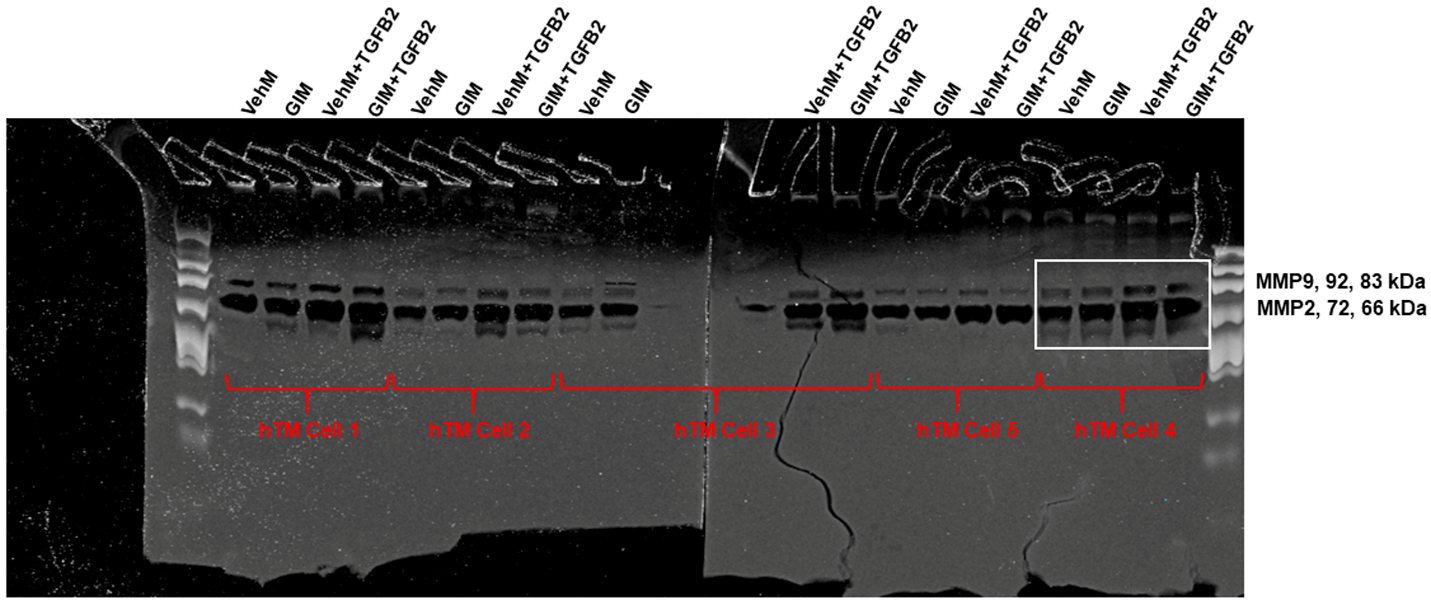


**Supplementary Figure S9.** Full length Gel Zymographs showing enzymatic activity of MMP2 and MMP9 in conditioned medium harvested from hTM cells that were seeded on VehMs or GIMs in the presence or absence of exogenous TGFβ2. Representative cropped samples (indicated by white box) are shown in Figure 8A and B.

**Supplementary Table S1. Antibodies for Western blotting analyses**

| **Antibody** | **Catalog Number** | **Dilution** | **Source** |
| --- | --- | --- | --- |
| Smad2 | 5339 | 1/2000 | Cell Signaling Technology, Danvers, MA, USA |
| Phospho-Smad2 | 18338 | 1/2000 |  |
| Smad3 | 9523 | 1/2000 |  |
| Phospho-Smad3 | 9520 | 1/2000 |  |
| P38 | 8690 | 1/5000 |  |
| Phospho-P38 | 4511 | 1/2000 |  |
| RhoA | 2117 | 1/5000 |  |
| ROCK1 | 4035 | 1/2000 |  |
| JNK | 9258 | 1/2000 |  |
| Phospho-JNK | 4668 | 1/2000 |  |
| CTGF | 86641 | 1/2000 |  |
| β-Actin | 4970 | 1/10000 |  |
| ERK1 | 610408 | 1/5000 | BD Biosciences, San Jose, CA, USA |
| Phospho-ERK1/2 | 612358 | 1/2000 |  |
| Αsma | A5228 | 1/5000 | Sigma-Aldrich, St. Louis, MO, USA |
| Goat anti-Rabbit IgG (H+L) Cross-Adsorbed Secondary Antibody, HRP | G-21234 | 1/10000 | Invitrogen, Carlsbad, CA, USA |
| Goat anti-Mouse IgG (H+L) Cross-Adsorbed Secondary Antibody, HRP | G-21040 | 1/10000 |  |

**Supplementary Table S2. Primers for qPCR**

| **GENE** | **FORWARD (5’-3’)** | **REVERSE (5’-3’)** |
| --- | --- | --- |
| **ECM structural genes** | | |
| FN1 | AATCCAAGCGGAGAGAGTCA | CATCCTCAGGGCTCGAGTAG |
| COL1A1 | GAGAGCATGAC-CGATGGATT | CCTTCTTGAGGTTGCCAGTC |
| COL4A1 | GGTATTCCAGGATGCAATGG | TCTCACCTGGATCACCCTTC |
| COL6A2 | AAGGAGAACCTGGGAGGAAA | GGTCCTGGGACTCCTCTTG |
| MYOC | TGTCCGCCAGGTTTTTGAGT | TGGAAATAGAGGCTCCCCGA |
| **Matricellular genes** | | |
| CTGF | ACCTGTGCCTGCCATTACAA | GCTTCATGCCATGTCTCCGT |
| SPARC | GTGCAGAGGAAACCGAAGAG | AAGTGGCAGGAAGAGTCGAA |
| TSP1 | ATGCTTATTTGTTCTCTACTGGCT | CTAAGCCTAGGCCTGAGCAAC |
| PAI1 | CCACTTCTTCAGGCTGTTCC | CCGTTGAAGTAGAGGGCATT |
| **ECM crosslinking genes** | | |
| LOX | CGACCCTTACAACCCCTACA | AAGTAGCCAGTGCCGTATCC |
| LOXL2 | CCTGGGGAGAGGACATACAA | CTCGCAGGTGACATTCTTCA |
| LOXL3 | CAACGCGGCCTTCTACAG | GGTGTCATTGGCACGATAGA |
| LOXL4 | CGACAGCCACTACTACAGGAAA | CTGGTGGATCCAGAAGGAGTT |
| TGM2 | CTCAGGGCTCACAGTGGAT | AGGGGTCCTATCTCTCATCCTG |
| **Genes responsible for ECM turnover** | | |
| MMP1 | CCAGGCCCAGGTATTGGAGGGG | GGCCGAGTTCATGAGCCGCA |
| MMP2 | ATAACCTGGATGCCGTCGT | AGGCACCCTTGAAGAAGTAGC |
| MMP9 | GAACCAATCTCACCGACAGG | GCCACCCGAGTGTAACCATA |
| MMP14 | CACCATGAAGGCCATGAGGC | GTATGTGGCATACTCGCCCA |
| ADAMTS4 | CATCCTACGCCGGAAGAGTC | TCTTGTCATCTGCCACCACC |
| TIMP1 | ACTACCTGCAGTTTTGTGGCT | CTGGTCCGTCCACAAGCAA |
| TIMP2 | AGGCTTAGTGTTCCCTCCCT | TGAGTGTGTCACCAAAGCCA |
| **Housekeeping gene** | | |
| GAPDH | GGTGAAGGTCGGAGTCAAC | CCATGGGTGGAATCATATTG |
